# Injury-specific factors in the cerebrospinal fluid regulate astrocyte plasticity in the human brain

**DOI:** 10.1038/s41591-023-02644-6

**Published:** 2023-12-08

**Authors:** Swetlana Sirko, Christian Schichor, Patrizia Della Vecchia, Fabian Metzger, Giovanna Sonsalla, Tatiana Simon, Martina Bürkle, Sofia Kalpazidou, Jovica Ninkovic, Giacomo Masserdotti, Jean-Frederic Sauniere, Valentina Iacobelli, Stefano Iacobelli, Claire Delbridge, Stefanie M. Hauck, Jörg-Christian Tonn, Magdalena Götz

**Affiliations:** 1 Chair of Physiological Genomics, Biomedical Center (BMC), Faculty of Medicine, LMU Munich, Planegg-Martinsried, Germany; 2 Institute of Stem Cell Research, Helmholtz Center München, Deutsches Forschungszentrum für Gesundheit und Umwelt (GmbH), Neuherberg, Germany; 3 Department of Neurosurgery, LMU University Hospital, LMU Munich, Munich, Germany; 4 Chair of Cell Biology, Biomedical Center (BMC), Faculty of Medicine, LMU Munich, Planegg-Martinsried, Germany; 5 SYNERGY Excellence Cluster of Systems Neurology, LMU Munich, Munich, Germany; 6 MediaPharma Srl, Chieti, Italy; 7 Department of Neuropathology, Institute of Pathology, TUM School of Medicine, TU Munich, Munich, Germany; 8 Research Unit Protein Science and Metabolomics and Proteomics Core, Helmholtz Zentrum München, Deutsches Forschungszentrum für Gesundheit und Umwelt (GmbH), Neuherberg, Germany

**Keywords:** Diseases of the nervous system, Neural stem cells

## Abstract

The glial environment influences neurological disease progression, yet much of our knowledge still relies on preclinical animal studies, especially regarding astrocyte heterogeneity. In murine models of traumatic brain injury, beneficial functions of proliferating reactive astrocytes on disease outcome have been unraveled, but little is known regarding if and when they are present in human brain pathology. Here we examined a broad spectrum of pathologies with and without intracerebral hemorrhage and found a striking correlation between lesions involving blood–brain barrier rupture and astrocyte proliferation that was further corroborated in an assay probing for neural stem cell potential. Most importantly, proteomic analysis unraveled a crucial signaling pathway regulating this astrocyte plasticity with GALECTIN3 as a novel marker for proliferating astrocytes and the GALECTIN3-binding protein LGALS3BP as a functional hub mediating astrocyte proliferation and neurosphere formation. Taken together, this work identifies a therapeutically relevant astrocyte response and their molecular regulators in different pathologies affecting the human cerebral cortex.

## Abstract (web summary)

Intracerebral hemorrhage triggers astrocyte proliferation and mediates neural stem cell potential via Galectin3 signaling.

## Main

Functional impairment is the main parameter to treat in any neurological disease, but most often there are few effective treatment options. To overcome this, a promising approach is to improve the function of glial cells, as they are important players not only in the intact brain for synaptic and circuitry functions, but also in pathological conditions where they regulate disease progression and scar formation^1–5^. A scar is dysfunctional tissue, and hence minimizing scar formation would improve neurological function. This is the case upon acute injury, such as traumatic brain injury (TBI), hemorrhage or stroke where the scar also often causes an epileptogenic region^3,4^, or in neurodegenerative disease, when glial cells determine the course of the disease (see, for example, ref. ^6^). Likewise, in other neurological diseases, such as glioma, the glial reaction can lead to epileptic dysfunction and determine tumor progression^7^. It is therefore essential to understand how to alter the glial environment to foster functional recovery and minimize disease progression. However, our knowledge about heterogeneity of glial response in human pathology is still in its infancy.

Preclinical studies suggest a beneficial role of proliferating astrocytes for functional post-injury recovery by reducing scar formation, helping to reseal the blood–brain barrier (BBB) and restraining monocyte invasion^2,8^. These proliferating astrocytes comprise a particularly plastic subset with neural stem cell (NSC) properties, upregulating NSC transcription factors after TBI^9,10^ and forming multipotent neurospheres^11,12^. They provide a source for neuronal replacement, as shown for example by deletion of Notch signaling^13^ or expression of neurogenic factors^14^. However, it is yet unknown if such a population that exerts beneficial functions for brain repair exists in the human brain. Typically, mostly GFAP immunoreactivity is examined in human pathology as it is increased in reactive astrocytes in response to different brain insults, including stroke, TBI, inflammation, tumors, epilepsy, Alzheimer’s disease, Parkinson’s disease and Huntington’s disease^1,6,15^. In this Article, we explored pathology-specific reactive astrocyte proliferation and acquisition of NSC hallmarks in the human brain.

## Results

### Reactive gliosis induced by cerebral cavernoma

**Fig. 1:**
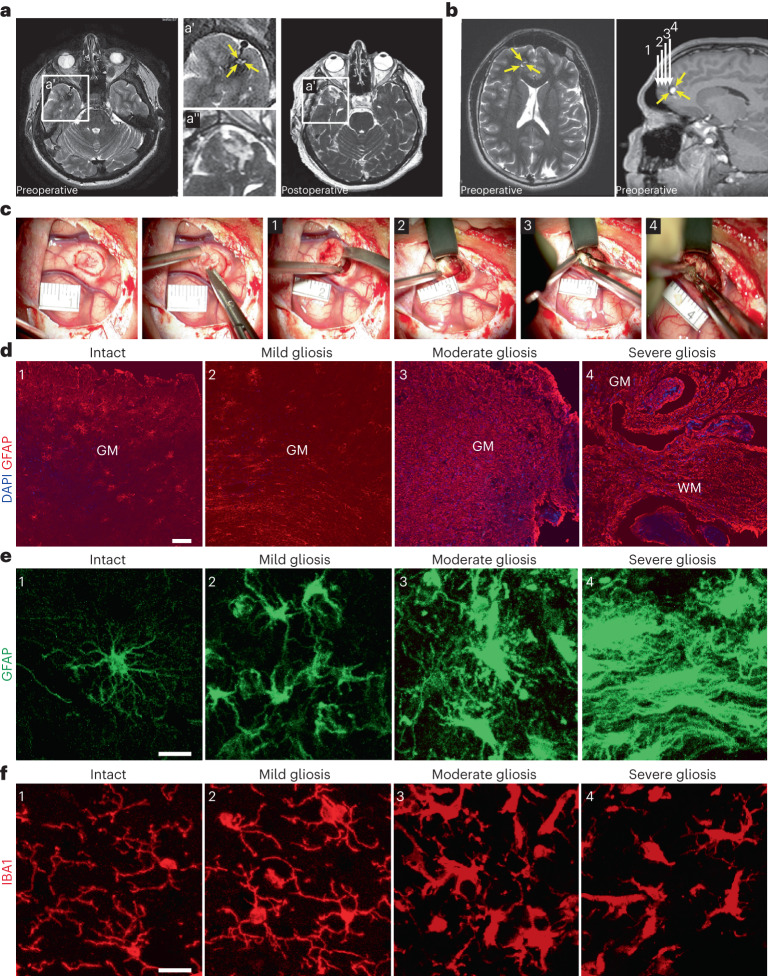
Progression of reactive gliosis with proximity to the CCM lesion. **a**,**b**, Representative MR T2-weighted axial (**a**, left panel in **b**) and T1-weighted sagittal (right panel in **b**) images obtained in patients with a solitary CCM lesion (yellow arrows) in the temporal (**a**) or frontal lobe (**b**). Preoperative images (at higher magnification **a′**) show typical ‘popcorn’ appearance of single CCM. Postoperative follow-up MRI (**a′** at higher magnification) without evidence of residual CCM confirms complete excision. White arrows (1–4 in **b**) point to the trajectory via which specimens were obtained. **c**, Intraoperative real-time image sequence showing the microscopic views of specimens sourced at corresponding position (1–4 in **b**). **d**, Representative images of GFAP immunoreactivity in regions 1–4. **e**,**f**, Note the manifestation of morphological changes in GFAP^+^ astrocytes (**e**) and IBA1^+^ microglia (**f**) with distance to the CCM core (as indicated by 1–4). Scale bars: 100 µm (**d**) and 20 µm (**e** and **f**).

Samples of the cerebral cortex from eight patients with sporadic supratentorial CCM (cerebral cavernous malformation) (Supplementary Table 1), undergoing surgical resection of nonneoplastic cortical tissue adjacent to the single hemorrhagic CCM lesion within the temporal (Fig. 1a) or frontal (Fig. 1b) lobes were acquired by the shortest possible trajectory from the brain surface to the CCM lesion, as indicated in the preoperative magnetic resonance imaging (MRI) in Fig. 1b. The intraoperative imaging allowed real-time delineation of pathology-affected tissue (into regions 2–4) well demarcated from the surrounding normal tissue (region 1) by a zone of gliosis or a rim of hemosiderin owing to the hemorrhagic CCM activity (Fig. 1c).

**Extended Data Fig. 1:**
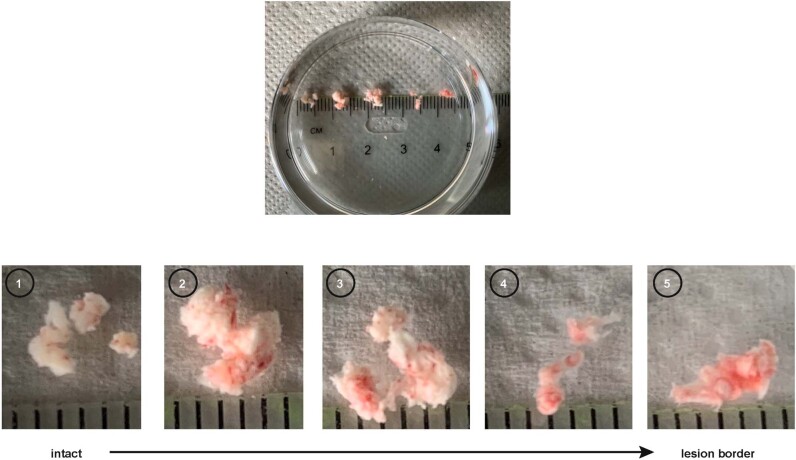
Example of research specimens collected from a patient with supratentorial CCM. Example of research specimens (1-5) contiguously harvested along the surgery trajectory from the brain surface to the CCM lesion core CCM. Each sample was then subdivided into two pair-blocks and used for immunohistochemistry and neurosphere-forming assay.

In each case, resected specimens contained the regions of cerebral cortex gray matter (GM) and white matter (WM) (Extended Data Fig. 1). The histopathological tissue status in regions 1–4 differed with distance from the CCM core, visible by changes in astrocyte morphology and intensity of GFAP immunoreactivity, reflecting a gradual increase in astrocyte reactivity closer to the CCM (Fig. 1d,e). The superficial GM tissue (region 1 in Fig. 1b,c) contained protoplasmic astrocytes with a characteristic bushy morphology and highly branched fine processes without any sign of cellular hypertrophy (Fig. 1e, position 1). In the deeper (but still superficial) GM parenchyma (region 2, where no abnormalities of signal-intensity were observed by T2-weighted MRI, Fig. 1b) few clusters of hypertrophic astrocytes were detectable (Fig. 1e, position 2). Astrocytes within the affected cortical areas (regions 3 and 4) with a pronounced T2W-hypointensity surrounding the ‘reticulated’ CCM core displayed hypertrophic morphology with processes in a dense meshwork reflecting a progressive increase from moderate (Fig. 1e, position 3) to severe astrogliosis (Fig. 1e, position 4) with increasing proximity to the CCM. Also, the density of astrocytes was highest in areas abutting the lesion core. Similarly, microglia showed a gradually more activated amoeboid or rod-like morphology closer to the lesion (Fig. 1f). As this was observed in all specimens, they were trichotomized into areas of mild, moderate and severe gliosis based on the morphology of astrocytes and microglia, while the samples without any sign of glial hypertrophy were deemed as intact, nonaffected.

### Cerebral cavernoma induces astrocyte proliferation

**Fig. 2:**
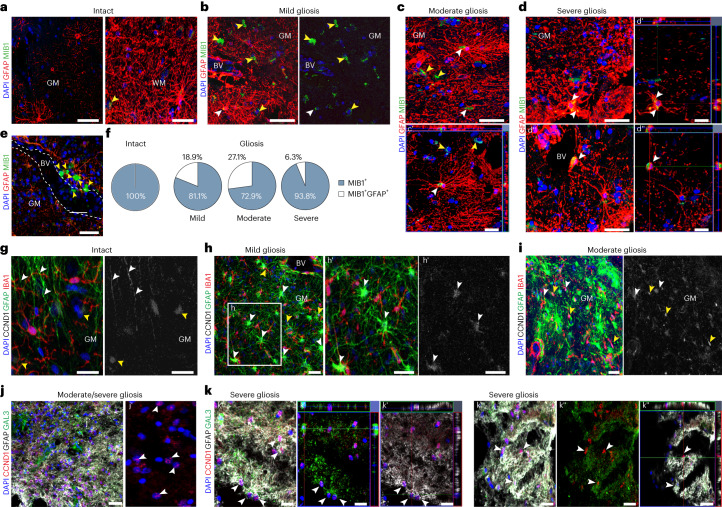
CCM rupture induces the astrocyte proliferation depending on the distance to the lesion site. **a**–**e**, Maximum intensity (**a**–**e**) and single optical projections (**c′**, **d′** and **d′′**) show representative examples of MIB1^+^ (yellow arrowheads) and MIB1^+^GFAP^+^ (white arrowheads) cells in the intact cerebral parenchyma (**a**) and in the regions of mild (**b**), moderate (**c**) and severe (**d**) gliosis. Note the accumulation of MIB1^+^ cells in the perivascular space or inside BV (dashed white lines) within mild gliotic parenchyma (**e**). **f**, Pie charts shown the mean proportion of GFAP^+^MIB1^+^ cells among the total MIB1^+^ population in the intact and gliotic regions of cerebral cortex (*n* = 4 patients). Appearance of CCND1^+^GFAP^−^ (yellow arrowheads) and CCND1^+^GFAP^+^ cells and processes (white arrowheads) within the upper cortical layers (**g**) and in regions of mild (**h**), moderate (**i**), moderate/severe (**j**) and severe (**k**) gliosis. Note that most CCND1^+^ astrocytes in moderate/severe gliotic area (**j** and **k**) are GAL3^+^ (white arrowheads). Orthogonal projections of the immunolabeled cells in **c**, **d** and **k** are shown in **c′**, **d′** and **d′′**, and **k′** and **k′′**, respectively. Scale bars: 50 µm (**a**–**e** and **g**–**j**), 25 µm (**h′** and **k**). Source data

**Extended Data Fig. 2:**
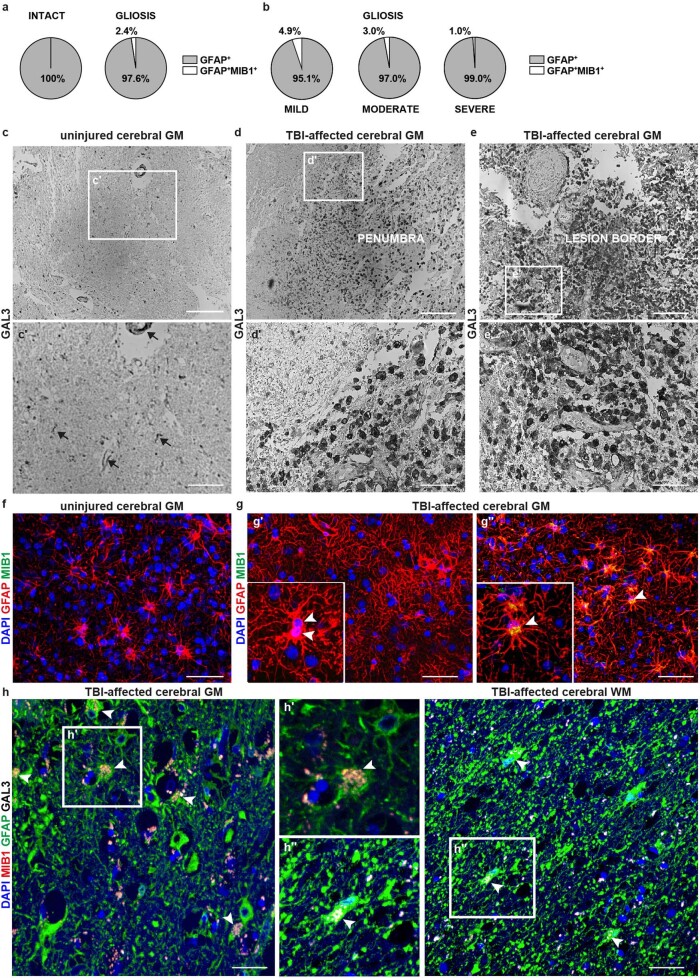
Astrocyte proliferation in the cerebral cortex from CCM or TBI patients. (***a***, ***b***) Pie charts showing the mean percentage of GFAP^+^MIB1^+^ cells among the total GFAP^+^ population in the intact and CCM-affected cerebral parenchyma and regions of mild, moderate and severe gliosis (n = 4 patients). (***c-e***) Immunohistochemical detection of GAL3 (based on the diamnobenzidine reaction) in postmortem samples of the cerebral cortex from TBI patients (n = 4). Higher magnification of boxed areas (c′e′) shows details of GAL3 immunoreactivity within the uninjured region (c), the penumbra (d) and directly at the border of the lesion (e). (***f***, ***g***) Representative single optical projections of GFAP and MIB1 immunolabelling in the intact (***f***) and TBI-affected (***g***) cerebral cortex (n = 3 patients). Note that some hypertrophic GFAP^+^ astrocytes are MIB1^+^ and divide within the gliotic GM (white arrowheads in inserts ***g***′, ***g***′′). (***h***) Maximum intensity projections show examples for GFAP, GAL3 and MIB1 immunolabeling in the TBI-affected cerebral GM (left panel) and WM (right panel) (n = 3 patients). Examples of MIB1^+^GFAP^+^GAL3^+^ cells are indicated by white arrowheads, and the boxed areas (***h′*** and ***h′′***) are shown at higher magnification. Further examples in Fig. 6 and Extended Data Fig. 9. BV: blood vessel, GM: cerebral grey matter, WM: cerebral white matter. Scale bars: 200 µm (c, d, e), 100 µm (c′, f, g), 50 µm (d′, e′, h). Source data

To examine the reactive astrocyte proliferation, GFAP-immunostaining was combined with the MIB1 antibody detecting the cell-cycle-associated protein Ki67 (ref. ^16^). In agreement with previous reports^17^, MIB1^+^ cells were virtually absent in the healthy cortical GM, and only very few were found in the intact WM (Fig. 2a). None of these sparse MIB1^+^ cells was GFAP^+^ (Fig. 2a), consistent with a quiescent state of astroglia in the intact human cerebral parenchyma. In contrast, the interindividual best-matched gliotic regions harbored a significantly higher number of parenchymal MIB1^+^ cells (0.7 ± 0.3 versus 12.8 ± 1.0 MIB1^+^ cells in the intact versus gliotic parenchyma, respectively; two-tailed *P* = 0.0056 from unpaired t test with Welch’s correction; *n* = 4 patients) (Fig. 2b–d). Many MIB1^+^ cells in the gliotic tissue were inside or surrounding blood vessels (BVs) (Fig. 2e), where proliferating endothelial cells, perivascular macrophages and/or inflammatory monocytes reside in context with the neurovascular abnormality in the CCM^18^. Among the parenchymal MIB1^+^ cells, GFAP^+^ cells reached more than a quarter of all proliferating cells in the moderate gliotic parenchyma (Fig. 2f), comprising 3% of astrocytes (Extended Data Fig. 2a,b). Notably, few MIB1^+^ reactive astrocytes were found at the border of the ruptured caverns, that is, in the severe gliotic region (Fig. 2d,f), where the entire MIB1^+^ cell population was substantially lower. Cyclin D1 (CCND1), that regulates progression through the restriction point at early G_1_ phase^19^, showed sparse expression in the intact cerebral parenchyma (Fig. 2g) but increasingly more immunostaining from mild (Fig. 2h) to severe gliosis (Fig. 2i–k) with particularly intensely labeled astrocytes in the moderate and severe gliotic parenchyma (Fig. 2i–k). There most CCND1^+^ hypertrophic astrocytes were GALECTIN3 (GAL3)^+^ (Fig. 2j,k), an inducer of CCND1 expression^20^. Thus, reactive astrocytes were MIB1^+^ and CCND1^+^ in the CCM affected regions, but close to the CCM core most cells were only CCND1^+^.

### GALECTIN3 marks proliferating astrocytes in cerebral cavernoma

**Fig. 3:**
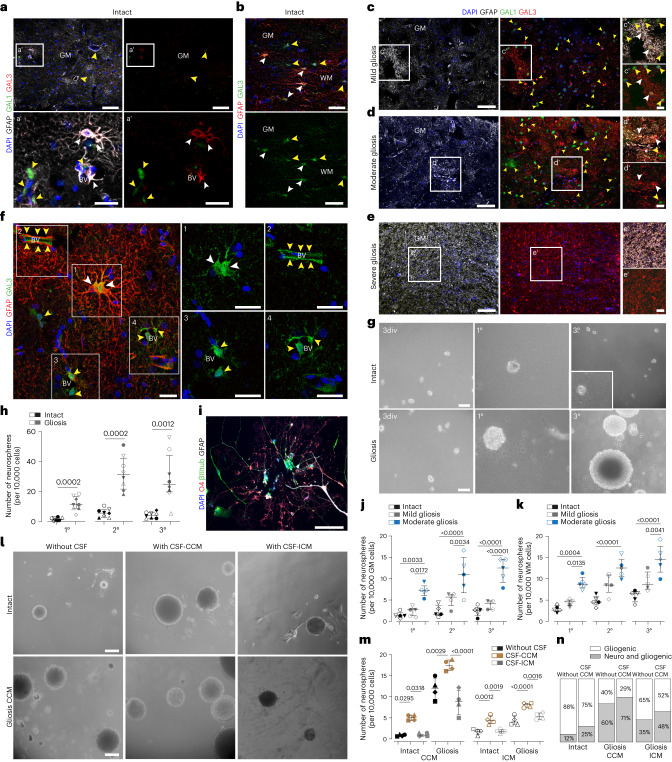
CCM induced astrocyte proliferation is accompanied by upregulation of GAL1/GAL3 and correlated with acquisition of NSC potential in vitro. **a**–**e**, Representative images of GFAP, GAL1 and GAL3 immunolabeling of intact GM (**a, a′**), WM (**b**) and CCM-induced mild (**c, c′**), moderate (**d**, **d′**) and severe (**e**, **e′**) gliosis. Yellow arrowheads indicate examples of GAL1^+^ cells; white arrowheads indicate examples of GAL3^+^GFAP^+^ cells. **f**, Representative image showing GAL3 immunostaining in GFAP^+^ dividing astrocyte (1), perivascular cells and at BV surface (2–4) within the gliotic parenchyma. Yellow arrowheads indicate Gal3^+^, GFAP-negative cells and outline BV; white arrowheads indicate of GAL3^+^GFAP^+^ cells. **g**,**h**, Examples of neurospheres derived from the intact or gliotic tissue samples (**g**) and the quantification of primary (1°), secondary (2°) and tertiary (3°) neurospheres generated after 14 d.i.v. (**h**). Data are presented as median and interquartile range. Each dot represents one patient. Two-sided *P* values from Mann–Whitney test. **i**, Representative image of βIII tubulin, GFAP and O4 immunostaining of differentiated neurosphere. **j**,**k**, The frequency of neurosphere-forming cells in the intact, mild or moderate gliotic GM (**j**) and WM (**k**). Data are shown as median with interquartile range (*n* = 5 patients per data group). Adjusted *P* values from one-way ANOVA followed by Tukey’s multiple comparison test. **l**,**m**, Disease-dependent CSF effects on sphere-forming capacity in human cortical cells obtained from CCM patients (**l**) and quantitative evaluation of these effects on the percent of neurospheres formed from 10,000 cortical cells from patients with CCM or ICM (**m**). Data in **m** are presented as median with interquartile range. Each dot represents one patient. Adjusted *P* values from one-way ANOVA followed by Tukey’s multiple comparison test. **n**, Neurospheres generated in the presence of CSF-CCM exhibit an increased neurogenic capacity. The frequency of neurogenic/gliogenic versus only gliogenic neurospeheres (*n* = 4 patients per data group per diagnosis). Scale bars: 130 µm (**c**–**e**), 100 µm (**a** and **b**), 50 µm (**g**, **i** and **l**) and 25 µm (**a′**, **c′**–**e′** and **f**). Source data

As both GAL1 and GAL3 regulate astrocyte proliferation in rodents^21,22^, we immunostained samples for these Galectins. The intact cortex showed little staining (Fig. 3a), and the few GAL1^+^ and/or GAL3^+^ cells were located along BVs. Except for a few juxtavascular astrocytes in the GM and rare astrocytes in the WM (Fig. 3a,b), astrocytes were GAL3 negative. GAL1^+^ cells exclusively located in the mild or moderate gliotic regions (Fig. 3c–e), while GAL3^+^ cells covered all regions of gliotic tissue (Fig. 3c–e). Reactive astrocytes became increasingly stronger GAL3^+^ closer to the CCM bleeding (Fig. 3c′,d′). As in the injured murine GM^21^, GAL3 (but not GAL1) was co-localized with GFAP (Fig. 3c–e), including double-positive astrocytes forming pairs (Fig. 3f1) (0–1 GFAP^+^GAL3^+^ pairs mm^−^^2^), reminiscent of proliferating astrocytes in the post-traumatic murine^21,23^ or human cerebral cortex (Extended Data Fig. 2) that stay closely together after cell division. Thus, in both murine and human cerebral cortex, GAL3 can be used as a new marker of proliferative reactive astrogliosis.

### Acquisition of NSC potential in cerebral cavernoma

Ki67 is high in fast-proliferating cells, while stem cells usually divide slowly and have undetectable Ki67 levels^24^. Probing which of these gliotic regions may contain cells with NSC potential, we cultured cells in neurosphere-forming conditions^11,12^. After 14 days in vitro (d.i.v.), cells from gliotic regions formed more than 10× higher number of neurospheres, while hardly any were detected in the cell cultures from intact cortical tissue (Fig. 3g,h). Neurosphere-forming cells were able to self-renew (Fig. 3h), reaching the same rate (35/10,000) as from the endogenous murine NSC niche^12^. They generated neurons, astrocytes and oligodendrocytes, when differentiated (Fig. 3i), thus fulfilling the NSC hallmark of multipotency. As neurosphere-formation of cells derived from the human WM had been previously reported^25^, we separated GM and WM in specimens from five patients before tissue dissociation and cultured cells derived from the GM and WM separately. After 14 d.i.v., the GM cells from regions of mild or moderate gliosis formed self-renewing neurospheres (Fig. 3j) in comparable numbers to WM-derived cells from analogous areas (Fig. 3k). In contrast, cells prepared from the nongliotic GM or WM tissue generated very few neurospheres, which did not expand in number after three passages (Fig. 3j,k). Taken together, CCM induces local NSC potential in the human brain parenchyma, with higher frequency of self-renewing neurosphere forming cells closer to the CCM core.

### Intracranial meningioma elicits distinct astrocyte reaction

**Extended Data Fig. 3:**
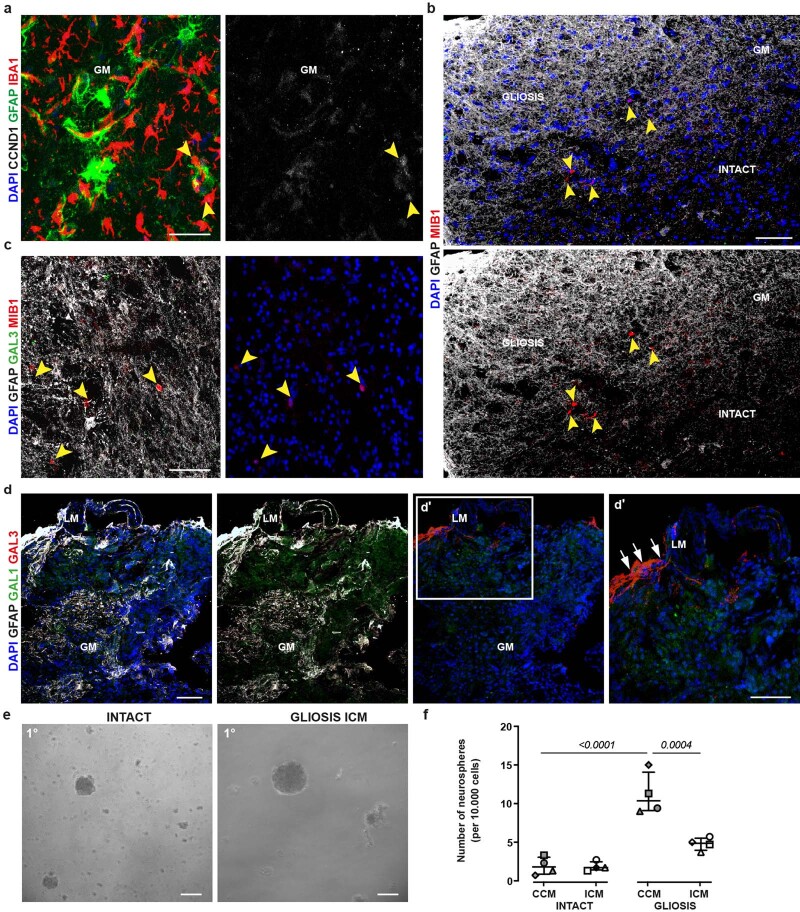
ICM-induced cerebral compression does not induce astrocyte proliferation and neurosphere formation. Representative images of GFAP immunolabelling in combination with CCND1 and IBA1 (***a***), MIB1 (***b***), GAL3 and MIB1(***c***) or GAL1/3 (***d***) in samples of cerebral cortex tissue from ICM patients (n = 4). Cell nuclei were counterstained with DAPI. IBA1^+^CCND1^+^ microglia are indicated by yellow arrowheads (*a*). Gliotic cerebral tissue surrounding ICM mass harbors few MIB1^+^ but GFAP^−^ cells (yellow arrowheads in *b, c)*. Note that GAL3 immunoreactivity is restricted to the leptomeninges (white arrows in ***d′***) which typically capsulated the noninfiltrating ICM tumor mass (higher magnifications of the boxed region in ***d′***) (n = 4 patients). Neurosphere-forming capacity of cells from intact or ICM affected gliotic cerebral tissue with example phase contrast micrographs (***e***) and quantification at 14 d.i.v. (***f***). Data are shown as median with interquartile range. Each dot represents one patient. Symbol shape and color indicates individual patient with CCM (grey) or ICM (empty). Adjusted *P*-values from one-way ANOVA followed by Tukey’s multiple comparison test. Data sets in f were used as part of analysis presented in Fig. 3m. GM: cerebral cortex grey matter, LM: leptomeninges. Scale bars: 100 µm (*b*, *c*), 70 µm (*a*), 50 µm (*d, e*). Source data

Given that noninvasive injuries fail to elicit astrocyte proliferation in mice^12^, we examined reactive astrogliosis in noninvasive benign intracranial meningioma (ICM). Tissue samples were obtained from four patients undergoing a total evacuation of the tumor mass directly attached to, but not infiltrated into the cerebral parenchyma. Similar to the CCM tissue samples, specimens obtained from patients with ICM contained fragments of nongliotic cerebral tissue as well as parenchyma with varying degree of gliosis due to the compression of adjacent tumor mass (Extended Data Fig. 3a,b). Despite profound gliosis in the tumor vicinity, there were very few MIB1^+^ and CCND1^+^ cells (Extended Data Fig. 3a–c). Consistent with previous observations^26^, GAL3 immunolabeling was restricted to leptomeningial cells surrounding the tumor mass (Extended Data Fig. 3d). Thus, ICM is not sufficient to elicit a proliferative response in astrocytes, as observed at the time of resection. Notably, cells dissociated from intact and gliotic cortical tissue from patients with ICM lacked neurosphere-forming capacity (Extended Data Fig. 3e,f). Thus, ICM stimulates neither the proliferation nor the dedifferentiation of reactive astrocytes, demonstrating the injury dependence of this phenotype.

### Pathology-dependent effects of CSF on neurosphere formation

Brain injury conditions affect the cerebrospinal fluid (CSF), and the CSF contains mitogenic signals^12,15^ that are necessary and sufficient to elicit reactive astrocyte proliferation and dedifferentiation in preclinical models^12^. Therefore, we collected subarachnoid CSF from patients with CCM (CSF-CCM) or ICM (CSF-ICM) to probe for effects on neurosphere formation. Intriguingly, the addition of CSF-CCM (100 μl ml^−1^) to neurosphere medium significantly increased neurosphere numbers and multipotency from either CCM-affected and unaffected areas of the same patients (Fig. 3l–n). Most strikingly, CSF-CCM, but not CSF-ICM, elicited significantly increased neurosphere formation also from cells of tissue samples resected during ICM evacuation (Fig. 3m). Moreover, the presence of CSF-CCM, but not CSF-ICM, further increased the proportion of multipotent neurospheres (typically generating 1–5% neurons), which was already higher in the cultures derived from the CCM-affected gliotic parenchyma than in those from patients with ICM (Fig. 3n). Together, these data suggested that the CSF of patients with CCM, but not patients with ICM, contains signaling molecules stimulating a NSC response.

### Proteome analysis of CSF from patients with CCM or ICM

**Fig. 4:**
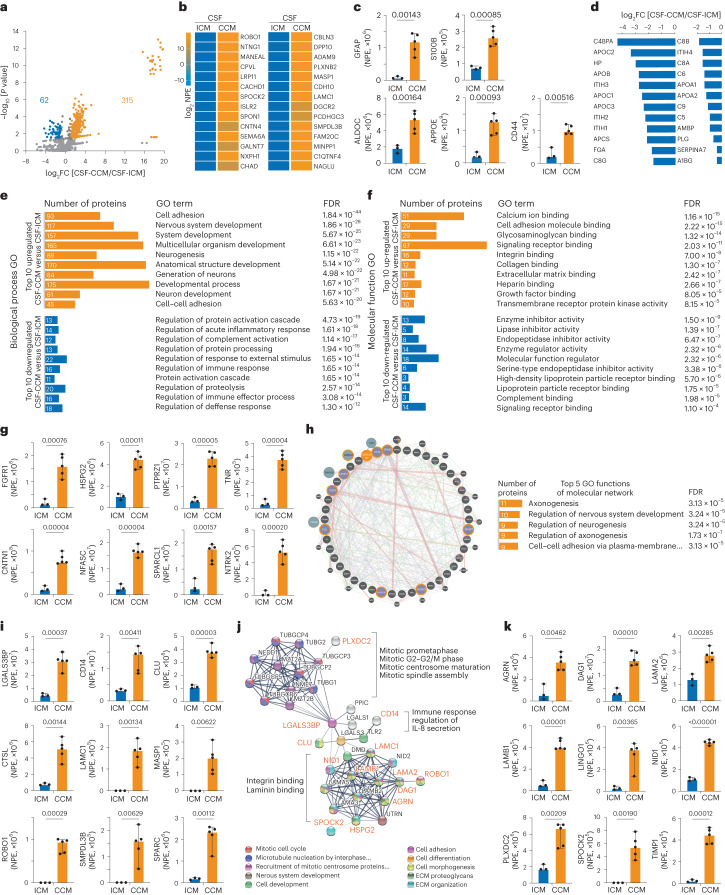
Disease-specific proteome profiles of subarachnoid CSF from patients with CCM or ICM. **a**, Volcano plot showing mean log_2_FC and the corresponding −log_10_ *P* values for 860 identified CSF proteins (*n* = 5 CSF-CCM, *n* = 3 CSF-ICM). Threshold of |−log_10_*P*| ≥1.3 and |log_2_FC| ≥1 was set to plot the statistically significant regulated proteins. Statistical significance from Bonferroni-corrected Welch t test of log transformed data. Proteins increased in CSF-CCM are highlighted in orange and those decreased in blue. **b**, Heatmap showing 28 proteins detected exclusively in CSF-CCM. **c**, NPE of astroglial markers in CSF-CCM and CSF-ICM. **d**, Levels of enrichment of 24 proteins implicated in meningioma development/progress. **e**,**f**, The top ten GO terms associated with significantly regulated proteins. **g**, NPE of significantly enriched CSF-CCM proteins regulating cell proliferation. **h**, Condensed interaction network of the top 45 most abundant significantly enriched (*P* ≤ 0.05, FC ≥ 3) proteins defining CSF-CCM signature (inner circle) with associated proteins (outer circles). LGALS3BP node in orange, LGALS3BP interactors in orange-delineated nodes. Colored lines denote the interaction type (Extended Data Fig. 6b–e and Source Data). **i**, NPE of LGALS3BP and its highly abundant interactors in CSF-CCM. **j**, PPI network of confirmed LGALS3BP interactions in *Homo sapiens* (STRING). Significantly upregulated CSF-CCM proteins in orange. **k**, NPE of in **j** highlighted LGALS3BP interactors. Data in **c**, **g**, **i** and **k** are presented as median ± 95% confidence intervals. Each dot represents one patient CSF sample (*n* = 3 patients with ICM and *n* = 5 patients with CCM). *P* values from unpaired *t*-test with Welch correction (FDR 1.00%). ECM, extracellular matrix; NPE, normalized protein expression. Source data

**Extended Data Fig. 4:**
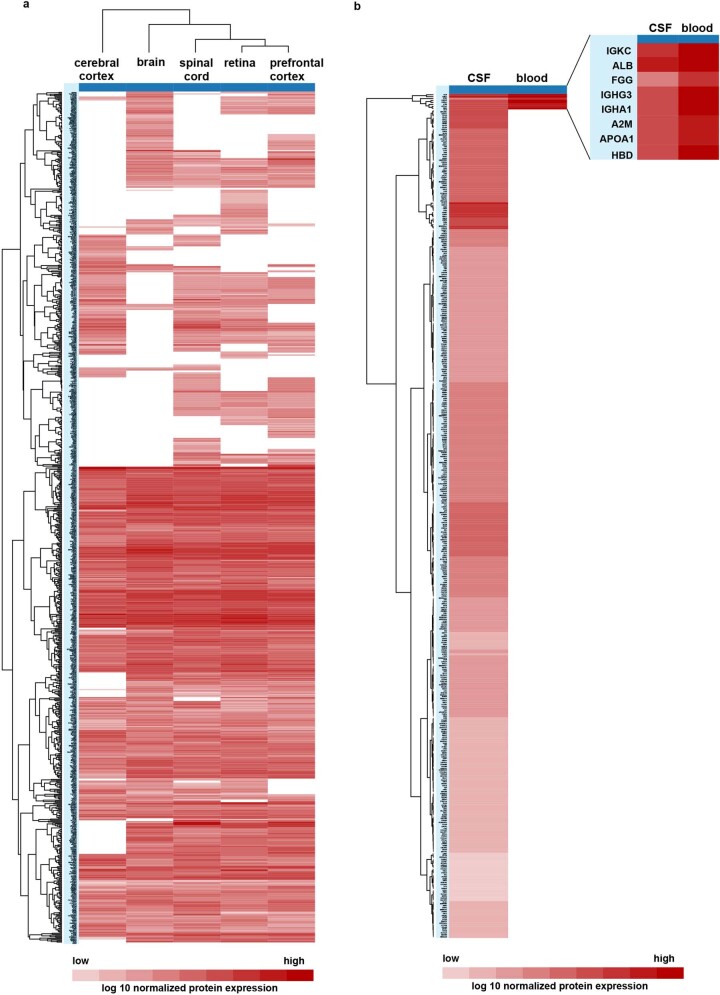
The proteins identified in CSF patient samples closely align with existing proteomics datasets of human neural tissue from different CNS regions and ‘pooled footprint’ of CSF. Heatmaps showing the 860 proteins identified across all our CSF samples and expressed as normalized values in different neural tissues (***a***), peripheral blood and CSF (***b***). Heatmaps were generated with the ProteomicsDB database analytic toolbox expression heatmap (by selecting MS1, Tissue, Fluid, and Swissprot only (https://www.ProteomicsDB.org)) and show the hierarchically clustered proteins in the selected biological sources.

**Extended Data Fig. 5:**
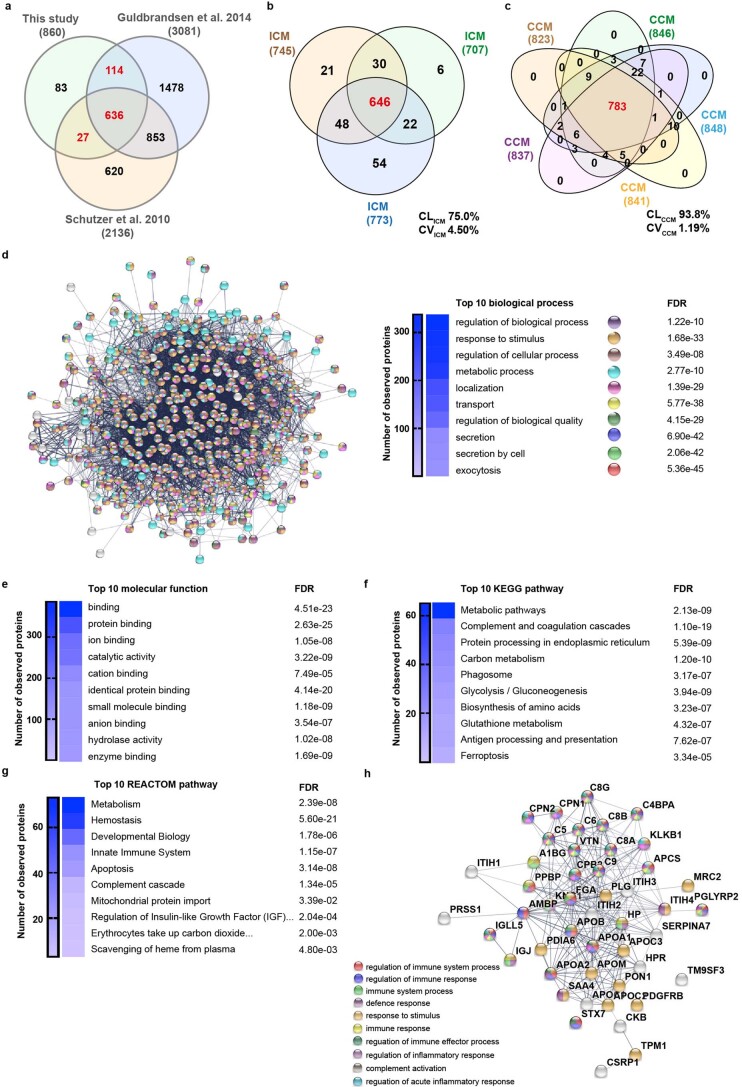
Comparison of CSF proteome datasets and Gene Ontology (GO) enrichment analyses. (***a***) *Venn diagram* showing the overlap between the 860 proteins (≥2 unique peptides and FDR < 0.01) identified in our study and two proteome datasets of CSF from neurologically healthy individuals published in Guldbrandsen et al., 2014 and Schutzer et al. 2010. (***b***, ***c***) Diagnosis-related overlap of proteome CSF profile in the 3 ICM patients (*b*) and 5 CCM patients (*c*). Only proteins that were found at least in one of three CSF-ICM samples or at least in two of five CSF-CCM samples were included. Interindividual variability between the CSF samples within each diagnosis group was calculated with confidence level (CL) for the 773 proteins in CSF-ICM samples (CL_ICM_ in *b*) and for the 848 proteins in CSF-CCM samples (CL_CCM_ in *c*). Coefficient of variation (CV) was defined as the ratio of the standard deviation (s) to the mean (x) in %: CV= s/x *100 (CV_ICM_ in *b*, CV_CCM_ in *c*) (***d-g***) The PPI network of 483 nonregulated (*P* > 0.05 and/or fold change <2 or >−2) proteins employed using STRING database, and the top 10 enriched GO terms for biological process (*d*) and molecular function (*e*), KEGG (*f*) and REACTOM pathways (*g*) are shown. The PPI network for the 62 significantly upregulated (*P* ≤ 0.05 and fold change ≥2) proteins in CSF-ICM versus CSF-CCM and the enriched biological process GO terms for these proteins are shown in ***h***. Source data

To identify similarities and differences in the proteomes of CSF-CCM and CSF-ICM, we used samples from five patients with CCM and three patients with ICM for quantitative label-free liquid chromatography–tandem mass spectrometry analysis identifying 860 proteins with ≥2 unique peptides and false discovery rate (FDR) <0.01 (Supplementary Table 2). Notably, the list of identified proteins closely aligns with existing human proteomics datasets of neural tissue from different regions of the central nervous system (CNS) and the ‘pooled footprint’ of CSF, but not peripheral blood (Extended Data Fig. 4). Moreover, ∼90% of proteins in our CSF samples overlap with published CSF datasets from healthy individuals^27,28^ (Extended Data Fig. 5a). The total number of detected proteins was similar within and between the two groups of CSF samples (coefficient of interindividual variation: CV_CCM_1.19%, CV_ICM_4.50%, CV_CCM-ICM_4.27%) (Extended Data Fig. 5b,c). About half of the identified proteins (56%; 483/860) were present at comparable levels in all CSF samples (Fig. 4a and Supplementary Table 3). This common CSF proteome showed Gene Ontology (GO) terms related to maintenance of various homeostatic functions (Extended Data Fig. 5d–g). Conversely, 377 proteins were significantly (fold change (FC) ≥2, *P* ≤ 0.05) different between CSF-CCM and CSF-ICM (Fig. 4a) with most (315) enriched in CSF-CCM (Supplementary Table 4) and only 62 enriched in CSF-ICM (Supplementary Table 5). Several differentially expressed proteins were diagnosis-confirming or reflecting the disease manifestation, for example, proteins associated with bleeding in the brain^29^ (CPVL, ISLR2, NXPH, FAM20, MASP1 and C1QTNF; Fig. 4b), predicted biomarkers of symptomatic CCM hemorrhage^30^ (for example, MMP2, CD14, PLXDC2, SPARC and IL6ST) or astroglial proteins (for example, S100B, GFAP, CD44, ALDOC and APOE) known for fast release into circulation after the BBB rupture^1,15^ detected exclusively enriched in CSF-CCM (Fig. 4c and Supplementary Table 4). Conversely, around 30% of proteins enriched in CSF-ICM were implicated in meningioma development or progression^31^, including the most common disease-monitoring markers in patients with meningioma APOA1, APOB, A1BG, HP, APCS and PDGFRB (Fig. 4d and Supplementary Table 5). As these data provide confidence in the CSF proteome composition, we next explored protein signatures that may contribute to eliciting astrocyte proliferation and NSC potential.

### Proliferation and stemness proteins only in the CSF-CCM

**Extended Data Fig. 6:**
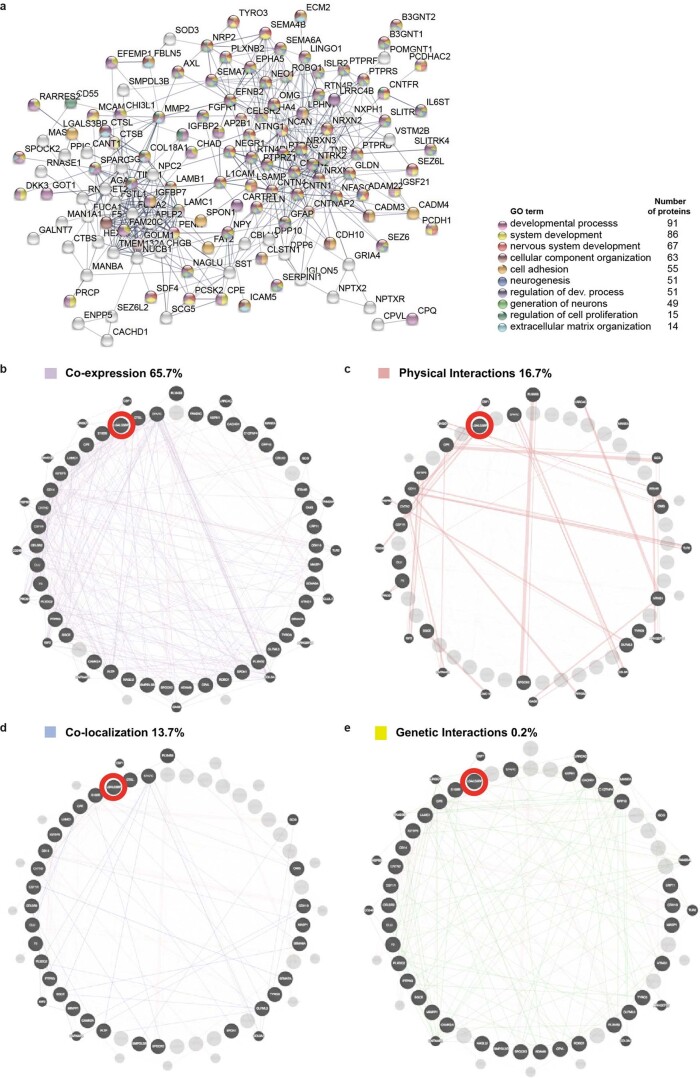
Network analysis of proteins significantly enriched in the CSF of CCM patients. (***a***) The PPI network generated using STRING database for the top 158 significantly up-regulated proteins (*P* ≤ 0.05 and fold change >5) in CSF-CCM versus CSF-ICM and the significantly enriched GO terms for biological process associated with these proteins. (***b-e***) Functional analysis of molecular networks for the 45 most abundant and significantly enriched (*P* ≤ 0.05 and fold change ≥3) proteins defining signature of CSF-CCM was performed using GeneMANIA v3.6.0 (Gene Function Prediction using a Multiple Association Network Integration Algorithm; www.genemania.org) and highlighted the relationships between these CSF-CCM proteins (nodes) connected (with edges) according to their co-expression, physical interactions, co-localization and genetic interactions from the database. Nodes in dark grey indicate interactors of LGALS3BP (red circle) and different line colors denote the type of these interactions. Source data

Interestingly, GO term analysis revealed ‘developmental process’, ‘nervous system development’ or ‘neurogenesis’ as significantly overrepresented processes in the proteins enriched in CSF-CCM (Fig. 4e,f and Supplementary Table 6), including regulators of the FGF-dependent NSC proliferation^32^ (FGFR1, HSPG2, PTPRZ1, TNR, NCAM1, NCAM2, CDH2 and L1CAM) with up to two orders of magnitude higher expression levels in CSF-CCM than in CSF-ICM (Fig. 4g and Supplementary Table 4) or modulators of IGFs (IGFBP2, IGFBP6 and IGFBP7) regulating NSCs^33^ significantly enriched in CSF-CCM (Supplementary Table 4). Protein–protein interaction (PPI) revealed proteins involved in the regulation of cell proliferation central in a STRING network of the 158 most enriched CSF-CCM proteins with two major hubs driven by IGF signaling or FGF2 pathway proteins (Extended Data Fig. 6a).

Conversely, 62 proteins enriched in CSF-ICM were associated with regulation of immune response, inflammation and complement activation (Fig. 4e,f), reflecting the ‘immunity block’ in preoperative patients with ICM^34^ that is represented by neutrophil inflammation, changes of platelet-lymphocyte ratio (Supplementary Table 7), the specific upregulation of the alternative complement cascade components^31^ (C5, C8A, C8B, C8G, C6 and C9; Fig. 4d) and the PPI network CSF-ICM-enriched proteins (Extended Data Fig. 5h) demonstrating a ‘pro-inflammatory’ CSF-ICM profile. These data provide the first evidence for the CCM-induced enrichment of CSF proteins involved in neurodevelopmental processes distinguishing its proteomic signature from the one of CSF-ICM.

### LGALS3BP as a novel biomarker of CCM in CSF

Next, we examined the most abundant and significantly upregulated proteins (FC ≥3, *P* < 0.05) in all CSF-CCM samples. Most of the 45 proteins that met these criteria (Supplementary Table 9) were related to ‘extracellular space’ or ‘integral components of plasma membrane’ (Supplementary Table 10) and implicated in various diseases or pathological processes within and outside the nervous system (Gene Set to Diseases, GS2D) (Supplementary Table 11). Notably, most of these proteins have not been previously identified in CCM profiling studies, and hence represent potentially novel CSF markers of this disease. The 45 proteins defining the CSF-CCM signature form a molecular network (Fig. 4h and Extended Data Fig. 6b–e) related to nervous system development, neurogenesis, axogenesis and cell–cell adhesion according to the GeneMANIA Human Database (see top five network functions in Fig. 4h). Among proteins enriched for interactions regulating these processes and associated with known CCM markers, we found LGALS3BP (Lectin galactoside-binding soluble 3 binding protein), an important clinical tumor biomarker^35^. LGALS3BP-interacting proteins, for example, ROBO1, were thousand-fold enriched in CSF-CCM compared with CSF-ICM (Fig. 4i). Interestingly, LGALS3BP appears functionally associated with the known CCM markers across multiple genotypes and species^30^, for example, PLXDC2, CD14 and SPARC (Fig. 4h and Extended Data Fig. 6), which were also significantly enriched in CSF-CCM (Fig. 4i–k). SPARC is expressed in human reactive astrocytes and modulates growth factor signaling and proliferation^36^, suggesting LGALS3BP as promising candidate to exert the functional effects of CSF-CCM. In further support, a STRING network of experimentally validated LGALS3BP interactions (Fig. 4j) highlighted a central role of this protein in translating multiple extracellular signals (including integrin/laminin binding that is profoundly dysregulated in the CCM^37^) into intracellular mechanisms driving mitotic activity (see annotations in Fig. 4j) of which several were indeed highly enriched in CSF-CCM (Fig. 4k).

### LGALS3BP in CSF-CCM promotes proliferation of human iPS cell-derived astrocytes

**Fig. 5:**
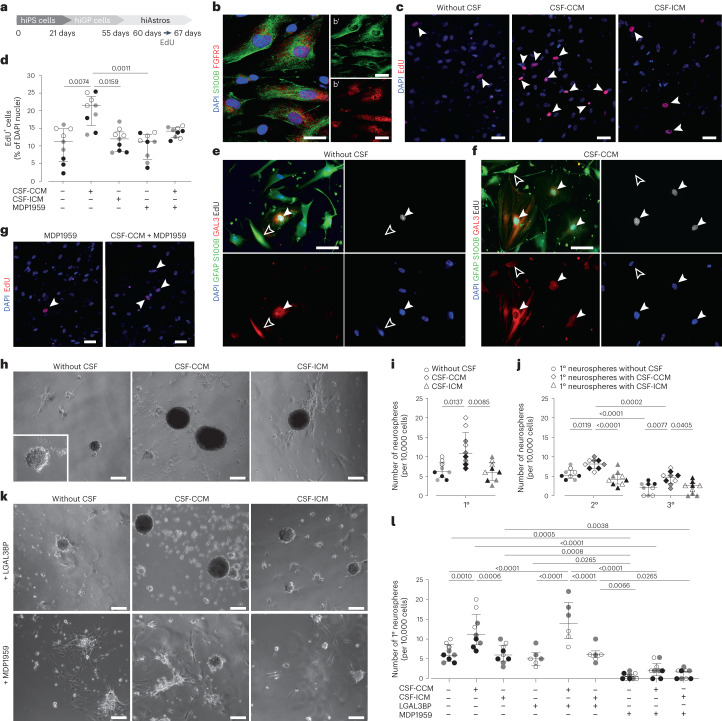
CSF-CCM promotes proliferation and NSC response in hiPS cell-derived astrocytes by LGALS3BP. **a**, Schematic timeline of experimental paradigm. **b**, Representative micrographs showing immunostainings for the astrocyte marker S100B and FGFR3 in hiAstros. **c**, Disease-specific CSF effects on EdU-incorporation in hiAstros cell cultures. White arrowheads indicate examples of EdU^+^ hiAstros. **d**, The percentage of EdU^+^ hiAstros in different culture conditions. **e**,**f**, Representative images of immunostaining for EdU, GFAP/S100B and GAL3 of hiAstros in control (**e**) or CSF-containing cultures (**f**). Filled white arrowheads indicate GAL3^+^EdU^+^ cells; empty white arrowheads depict Gal3^+^EdU^−^ astrocytes. **g**, Representative micrographs showing reduced hiAstros proliferation after exposure either to LGALS3BP-function blocking antibody MDP1959 alone or in combination with CSF-CCM. **h**,**i**, Phase-contrast images of primary neurospheres generated from hiAstros in control or CSF-containing cultures (**h**), and the percent of neurospheres formed from 10,000 hiAstros in these culture conditions (**i**). **j**, Self-renewal of neurospheres shown as number of neurospheres formed after passages (2° and 3°, secondary and tertiary neurospheres). **k**,**l**, The effects of rhLGALS3BP or MDP1959 treatment on neurosphere-forming capacity of hiAstros with example pictures (**k**) and quantification (**l**). Data in **d**, **i**, **j** and **l** are presented as median and interquartile range. Each dot represents one biological replicate per iPS cell line. Symbol indicates iPS cell line: #1 (empty), HMGU12 line (gray) and UKERi82a-R1-002 (black). Adjusted *P* value from Kruskal–Wallis test with Dunn’s for multiple comparisons test (in **d** and **i**), an ordinary one-way ANOVA followed by Holm–Šídák’s multiple comparisons test (in **j**), Brown–Forsythe ANOVA with Tukey’s multiple comparisons test (in **l**). hiGP, human induced glial progenitor. Scale bars: 50 µm (**h** and **k**), 25 µm (**c**, **e**, **f** and **g**) and 15 µm (**b**). Source data

**Extended Data Fig. 7:**
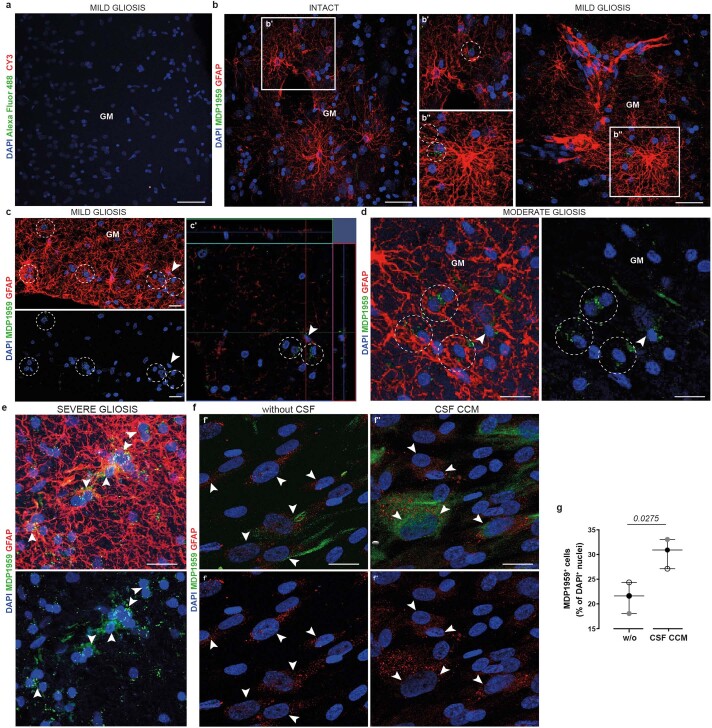
Expression of LGAL3BP in the human cerebral parenchyma and in human IPSC derived astrocytes. (***a***) Representative maximum intensity projection of a human cerebral cortex section showing the absence of immunostaining when the primary antibodies against GFAP and LGALS3BP (MDP1959) were omitted from the primary staining step in immunodetection protocol (n = 4 patient samples). (***b***) Immunostaining for GFAP and MDP1959 (detecting LGALS3BP) showing a sparse labelling in the intact cerebral parenchyma (*b*′) and in the gliotic region (*b*′′) distal to the hemorrhagic brain lesion. Boxed areas in *b*′ and *b*′′ are shown at higher magnification. Circles indicate the areas of MDP1959 immunostaining (n = 4 patients). Maximum intensity (***c***) and orthogonal projection (*c’*) images showing the examples of LGALS3BP immunolabelling (dashed circles) in the gliotic regions and few LGALS3BP^+^GFAP^+^ cells (white arrowheads) (n = 3 patients). GFAP^+^LGALS3BP^+^ reactive astrocytes in regions of moderate (***d***) and severe gliosis (***e***) are indicated by white arrowheads (n = 4 patients). (***f***) Single optical plane images showing representative examples of LGALS3BP^+^ hiAstros (white arrowheads) cultured under control condition (*f*′) or in the presence of CSF-CCM (*f*′′). Histogram depicting the percentage of LGALS3BP^+^ hiAstros in the control and CSF-CCM containing cell culture medium (***g***). Data are shown as median with interquartile range. Each dot represents one biological replicate (n = 3 per biological replicate). Dot color indicates iPSC line: #1 (empty), HMGU12 (grey), UKERi82a-R1-002 (black). Two-tailed *P*-value from unpaired t-test with Welch’s correction. Scale bars: 50 µm (*a*, *b*), 30 µm (*c*), 20 µm (*d, e*), 15 µm (*f*). Source data

To examine LGALS3BP function, human induced pluripotent stem (hiPS) cells were differentiated in human induced astrocytes (hiAstros) for 60 days (Fig. 5a). hiAstros displayed many characteristics of astrocytes, including FGFR3 immunostaining (Fig. 5b), and most (∼90%) were quiescent, not incorporating 5′-ethynyl-2′-deoxyuridine (EdU) added for 7 days at day 60 of differentiation (Fig. 5a,c,d). In the presence of CSF-CCM, but not CSF-ICM, hiAstros EdU incorporation doubled (Fig. 5c,d). Of note, EdU^+^ hiAstros were also GAL3^+^ (Fig. 5e,f), and the frequency of GAL3^+^ hiAstros significantly increased by CSF-CCM (23 ± 1.7% versus 34 ± 3% in control versus CSF-CCM cultures; two-tailed *P* = 0.0083 from unpaired t test; *n* = 3). As this assay provided a readout for CSF-CCM on human astrocyte proliferation, we probed the functional role of LGALS3BP by adding MDP1959, a humanized version of anti-LGALS3BP antibody (from MediaPharma)^38^ that also detects LGALS3BP in reactive astrocytes of the hemorrhage-affected human cerebral parenchyma (Extended Data Fig. 7a–e) and in hiAstros (Extended Data Fig. 7f,g). Indeed, this antibody could block the effect of CSF-CCM on hiAstros proliferation (Fig. 5d,g).

### LGALS3BP in CSF-CCM promotes neurosphere formation

Next, we used the neurosphere formation of hiAstros as a readout. CSF-CCM, but not CSF-ICM, significantly increased formation of self-renewing neurospheres (Fig. 5h–j). This was almost completely blocked by addition of the MDP1959 antibody (Fig. 5k,l), demonstrating a key role of LGALS3BP in the CSF-CCM in this response. To probe if this response could further be increased, purified recombinant human (rh) LGALS3BP (10 μg ml^−1^) was added alone or in combination with CSF-CCM or CSF-ICM (Fig. 5k,l). Remarkably, CSF-CCM and rhLGALS3BP co-treatment resulted in three-fold increased number of neurospheres, while rhLGALS3BP alone or in conjunction with CSF-ICM had no effect (Fig. 5k,l), not even in the double concentration (3.8 ± 0.5 versus 4 ± 1.0 spheres in the cultures with 10 μg versus 20 μg rhLGALS3BP per milliliter of medium; two-tailed *P* = 0.8601 from unpaired t test with Welch’s correction, *n* = 3). Moreover, rhLGALS3BP addition to the CSF-CCM did not significantly increase neurosphere formation compared with CSF-CCM alone, suggesting that levels of LGALS3BP in the CSF-CCM are close to saturating. Taken together, we identified the LGALS3BP–GAL3 axis as a key regulator of astrocyte proliferation and neurosphere formation.

### GALECTIN3^+^ proliferating astrocytes only upon hemorrhage

**Fig. 6:**
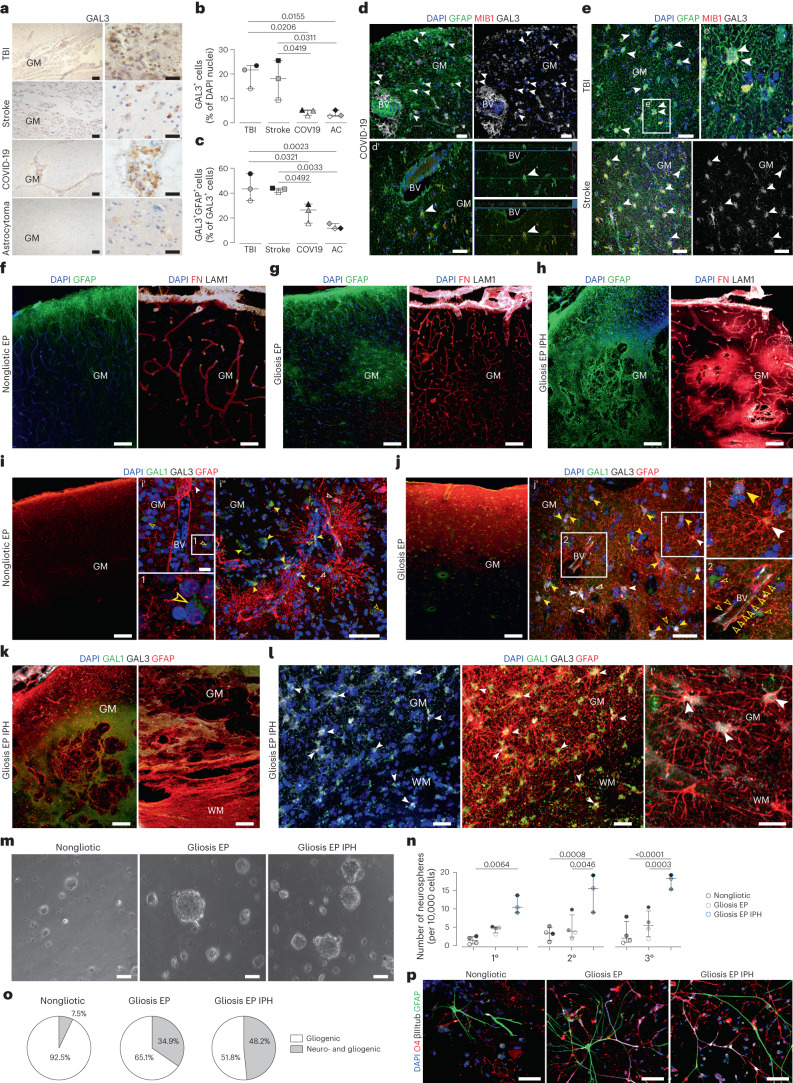
Pathology-specific GAL3 expression in human reactive astrocytes is tightly correlated with their proliferative response and neurosphere-forming capacity. **a**, Representative GAL3 immunolabeling (brown color) of gliotic cerebral cortex from the patients with TBI, stroke, COVID-19 or AC (higher magnifications in right panels). **b**,**c**, Proportions of GAL3^+^ (**b**) and GFAP^+^GAL3^+^ cells in these pathologies (**c**). **d**, Appearance of GAL3^+^GFAP^+^ cells and examples of GAL3^+^GFAP^+^MIB1^+^ astrocytes indicated by white arrowheads (**d**, **d′**) within the edematous GM from COVID-19 victim. **e**, Micrographs of GAL3^+^MIB1^+^GFAP^+^ cells (white arrowheads) in the TBI or stroke affected GM (*n* = 3 sections per patient per diagnosis in **a**–**e**). **f***–***h**, Images of GFAP (left) and FN/LAMININ1 (LAM1, right) immunolabeling in the nongliotic (**f**), gliotic (**g**) and hemorrhage-affected gliotic (**h**) cerebral tissue from patients with epilepsy (EP). **i***–***l**, Examples of GAL1^+^GFAP^−^ or GAL3^+^GFAP^−^ (empty yellow arrowheads), GAL1^+^GAL3^+^GFAP^−^ (yellow arrowheads) and GAL3^+^GFAP^+^ (white arrowheads) cells in nongliotic (**i, i′**), gliotic (**j, j′**) and (micro)hemorrhage-affected (**k** and **l**, **l′**) tissue samples from patients with epilepsy. Phase-contrast examples of neurospheres (**m**) formed from tissue from patients with epilepsy (*n* = 4) and their quantification over three passages for self-renewal (**n**). **p**,**o**, Fluorescence micrographs (**p**) show their differentiation as quantified (**o**). Data are presented as median and interquartile range (**b**, **c** and **n**). Each dot represents one patient. Adjusted *P* values from ordinary one-way ANOVA followed by Tukey’s multiple comparisons test (**b**, **c** and **n**). Scale bars: 100 µm (**f**–**k**), 75 µm (**a**, **d** and **i′**), 50 µm (**e**, **j′**, **l**, **m** and **p**) and 25 µm (**l′** and **p**). Source data

**Extended Data Fig. 8:**
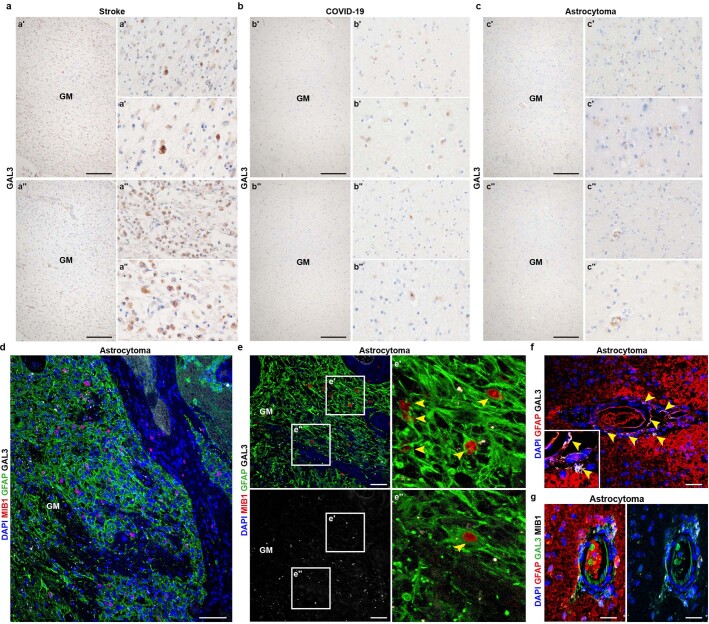
Diagnosis-related expression of GAL3 in the human cerebral cortex. (***a***-***c***) Appearance of GAL3 (brown color) in individual autopsy samples of the cerebral cortex parenchyma from patients with stroke (*a,a*′, *a*′′), COVID-19 (*b, b*′, *b*′′), as well as in biopsy samples of astrocytoma (c, *c*′, *c*′′). Examples of the affected cerebral GM areas (left panels in ***a***-***c***) are shown at higher magnifications (right panels in ***a***-***c***). (***d***) Representative maximum projection image showing examples for GFAP, GAL3 and MIB1 immunolabelling in astrocytoma biopsy (***e***-***g***). Notably, GFAP^+^MIB1^+^ cells in tumor adjacent tissue are GAL3^−^(yellow arrowheads in *e*′ and *e*′′), while proliferating (MIB1^+^) cells at the pathological tumor vessels express GAL3 (***f***, ***g***). n = 3 patients per diagnosis. GM: cerebral grey matter. Cell nuclei were counterstained with hematoxylin (in *a-c*) or DAPI (in *d*-*g*). Scale bars: 150 µm (*a-c*), 75 µm (*d*, *e*), 50 µm (*f*), 20 µm (*g*).

Given the above identification of GAL3^+^ proliferating astrocytes in CCM with intracerebral hemorrhage, but not in patients with ICM without it, we asked if this may be special for CCM, or a more generally applicable rule. We therefore examined individual biopsy and autopsy samples of human cerebral tissue with prominent intracerebral hemorrhage due to TBI, stroke or without, as upon severe acute respiratory syndrome coronavirus 2 infection (coronavirus disease 2019, COVID-19) or astrocytoma (AC, grade 2/3) carcinogenesis (Fig. 6a and Extended Data Figs. 2 and 8). In all cases (three patients per diagnosis) we detected GAL3^+^ cells (Fig. 6a and Extended Data Figs. 2 and 8). Remarkably, their number differed significantly between the diseases, highest in the TBI- or stroke-affected parenchyma, and very low in AC or COVID-19 specimens (Fig. 6b). Also, the number of GAL3^+^GFAP^+^ astrocytes was significantly increased in samples from patients with TBI or stroke compared with COVID-19 or AC (Fig. 6a,c–e). In the COVID-19 cases, most GAL3^+^GFAP^−^ cells were predominantly located along leptomeningeal or cortical BVs (Fig. 6a,d), probably related to the hyperinflammatory syndrome^39^. This is relevant, as in two of three COVID-19 cases we observed GFAP^+^GAL3^+^ cells accumulating close to enlarged perivascular spaces (Fig. 6d,d′) indicative for vasogenic edema due to leakage of blood products. This may explain the slightly higher rate of GAL3^+^ astrocyte proliferation (5.4% of all GFAP^+^ cells) in the neocortex exhibiting cerebrovascular edema (Fig. 6d), which was still half of the proportion observed in stroke (10.4%). Notably, there were very few proliferating (1.9% of all GFAP^+^) reactive astrocytes in COVID-19 specimens with no obvious signs of edema.

Triple immunostaining for MIB1, GFAP and GAL3 confirmed reactive astrocyte proliferation (Fig. 6d,e) with 4.4% MIB1^+^ among GFAP^+^ astrocytes in TBI at comparable levels to the mild and moderate gliosis in the CCM condition (Fig. 6e and Extended Data Fig. 2g). Stroke samples had a substantially higher proportion of proliferating astrocytes (10.4%) (Fig. 6e). Thus, the proliferative astrocyte response to the perilesional hemorrhage in CCM samples is not maximal, and can further increase, such as upon stroke.

**Extended Data Fig. 9:**
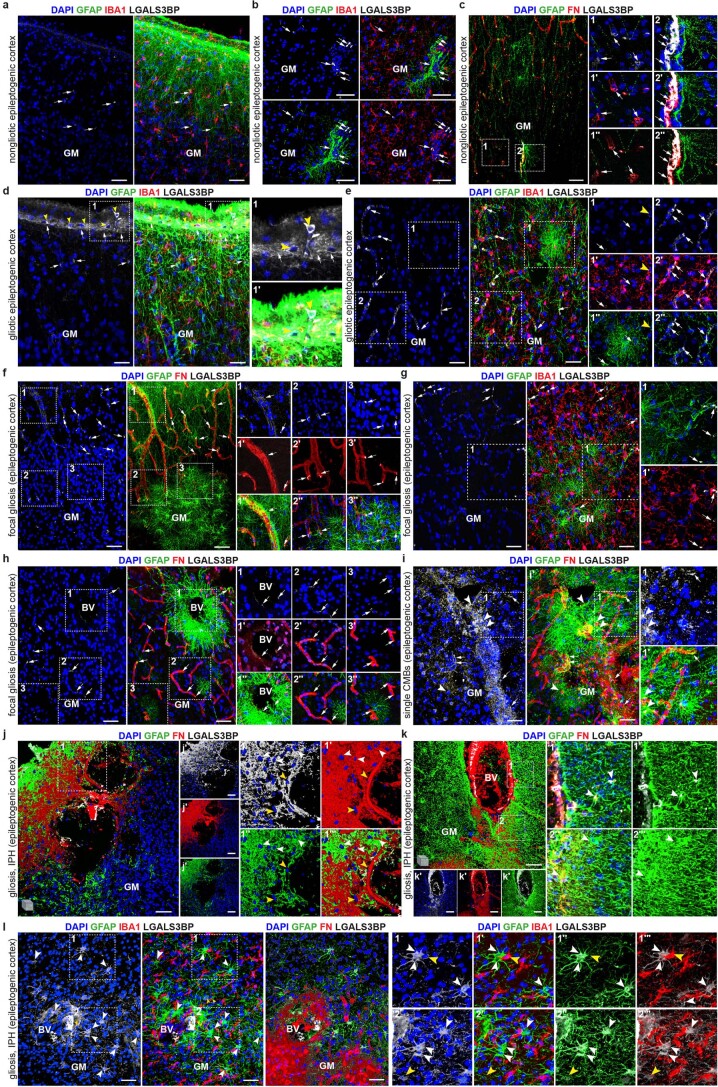
(***a***, ***b***) Representative GFAP, IBA1 and LGALS3BP immunolabelling in the nongliotic cerebral cortex areas from epilepsy patients (n = 5). Note the LGALS3BP staining in unaffected parenchyma is mainly associated with the BV (white arrows), as demonstrated by co-labeling with FIBRONECTIN (FN) (***c***). Representative images of immunostaining for GFAP, LGALS3BP and IBA1 (***d***, **e**) or FN (***f***) in gliotic epileptogenic cortex and region with focal gliosis (***g***, ***h***). White arrows indicate LGALS3BP expression in vicinity of the BV. Yellow arrowheads indicate LGALS3BP^+^ microglia (IBA1^+^) and leptomeningial cells (n = 5 patients). Representative images of immunostaining for LGALS3BP, GFAP and FN in gliotic areas with single CMB (***i***) or intraparenchymal hemorrhage, IPH (***j***, ***k***). (n = 4 patients). Note the accumulation of LGALS3BP^+^GFAP^−^ cells (yellow arrowheads) and LGALS3BP^+^GFAP^+^ (white arrowheads) in the vicinity of disrupted vascular walls (*j*, *k*). (***l***) Appearance of LGALS3BP^+^ hypertrophic reactive astrocytes (white arrowheads) and microglia (yellow arrowheads) within hemorrhage-affected cerebral parenchyma. Single optical sections (*l*1 and *l*2) shown an example of LGALS3BP^+^GFAP^+^ dividing astrocytes (white arrowheads) (n = 4 patients). Dashed white boxes in (*c*, *d*-*l*) are shown at higher magnification. 3D-view of maximum projection images are shown in *j* and *k*. Cell nuclei were counterstained with DAPI. BV: blood vessel, CMB: cerebral microbleeding, GM: cerebral grey matter, IPH: intraparenchymal hemorrhage. Scale bars: 100 µm (*c, j, j*′*, k, k’*), 50 µm (*a, b, d-l*).

Given that most common etiologies for acquired epilepsy (in consequence of brain insults, including TBI, stroke, acute infections and cancer) are associated with BBB opening^40^, we explored surgical samples from five patients with epilepsy (Supplementary Table 1). No GAL3^+^ astrocytes were present in nongliotic or focal gliotic epileptogenic cortex (Fig. 6f,g,i,j), while pronounced GAL3 immunoreactivity (Fig. 6k) and many GAL3^+^ astrocytes (Fig. 6l) were present in regions with intraparenchymal hemorrhage (IPH) detected by large fibrinogen (FN)-immunopositive patches (Fig. 6h). Likewise, LGALS3BP immunostaining also appeared correlated with severity of the gliosis and in FN-positive patches (Extended Data Fig. 9). Thus, in several pathologies and in intrapatient comparison the correlation between hemorrhage and GAL3^+^ astrocyte pertains.

### Neurosphere formation from tissue of epileptogenic regions

To explore if also neurosphere formation correlates to hemorrhage, we cultured cells from nongliotic, gliotic and gliotic with IPH regions of epileptogenic cortex (Fig. 6m). At all passages there were significantly more neurospheres formed by IPH-affected regions than other tissue samples (Fig. 6n). Cells from gliotic regions without IPH generated as few self-renewing neurospheres as nongliotic tissue visible after several passages (Fig. 6n), similar to tissue from patients with ICM. Upon differentiation, neurospheres from nongliotic tissue comprised only 7% multipotent spheres, which was higher in gliotic tissue (35%) and peaked in the gliotic with IPH tissue samples (51%) (Fig. 6o,p). Thus, half of all the neurospheres were multipotent NSCs with the capacity for self-renewal. These data demonstrated that the NSC response is not special for CCM but is also present in the frequent condition of epilepsy with IPH.

## Discussion

Much has been learned in recent years about the importance of the local environment and glial cells for disease progression and functional recovery^3,6,10,41–43^. Here we discovered intracerebral hemorrhage as common denominator for eliciting reactive astrocyte proliferation and GAL3 upregulation in a wide range of pathologies. This proliferative astrocyte reaction takes place in cerebral cavernoma, stroke and TBI samples, while it is virtually absent in meningioma, COVID-19 or the AC surrounding parenchyma. Even within the samples from the same epilepsy patient GAL3^+^ astrocytes were found in regions with, but not without, microbleedings. The comprehensive proteome of the CSF of two conditions (CCM and ICM) revealed a dedifferentiation response upon brain hemorrhage and a wealth of possible biomarkers for this condition. This will now allow clinical stratification of this reactivity aligning it with disease progression. Beyond this, we identified LGALS3BP as a crucial functional regulator of this response in the CSF. Blocking LGALS3BP in the CSF-CCM blunted the astrocyte proliferation and neurosphere formation response, highlighting the key role of this protein in initiating astrocyte proliferation and NSC properties. Taken together, our data provide a general mechanism, intracerebral hemorrhage, and a molecular regulator, LGALS3BP, instructing a functional astrocyte subset with high therapeutic potential.

### Diagnosis and relevance of astrocyte proliferation

Here we discovered disruption of BBB integrity as a common trigger for the proliferation of human parenchymal astrocytes and the emergence of NSC-like cells. Despite the shared histopathological characteristics, such as cytoskeletal hypertrophy and GFAP upregulation, astrocytes activated by compression, infection or hyperexcitation failed to initiate a proliferative program. This finding fits well with previous data in mice^11,23^.

But why is it important to know about astrocyte proliferation triggered by cerebral hemorrhage? Firstly, more and more case reports link previous TBI with increased rates of glioma emergence (for review see ref. ^7^), and proliferating astrocytes are a likely origin for this. Also epilepsy or mood disorders following stroke or TBI have been linked to astroglia abnormalities^41,43^, as disease progression in Alzheimer’s disease^6^ or chronic pain^42^. Indeed, proliferating astrocytes have specific molecular properties, as highlighted in previous preclinical mouse models^9,12,21^ and here by GAL3 expression. Moreover, proliferating astrocytes are predominantly located at the BVs^8,23^ and influence the invasion of monocytes and scar formation. Conversely, the scar is what causes new epileptic foci due to lack of inhibition and reduced glutamate transporters^4^. Thus, identifying the presence of this specific functional astrocyte subtype in an entire group of very frequent brain pathologies is a major step forward towards exploiting their beneficial roles or stratifying the glial reactivity in relation to disease progression.

### GALECTIN3 as marker and functional player in astrogliosis

Notably, GAL3 in astrocytes was associated with BBB rupture in CCM, TBI, stroke and epilepsy with (micro)bleedings, but not present in epilepsy samples without FN invasion, patients with COVID-19 or stroma surrounding meningioma or glioblastoma. This Galectin is garnering clinical interest as biomarker for different pathophysiological processes^22^ and serves as a predictor of the severity and clinical outcome in a variety of brain lesions, including trauma, stroke and hypoxic ischemia^22^. HIF1α mediates GAL3 upregulation in response to hypoxic conditions^20,22^. In the CCM-mediated mild hypoxia release of nitric oxide from dysfunctional endothelium can reach cells millimeters away to activate HIF1α in astrocytes within CCM tissue^44^, as upon stroke or TBI, where hypoxia also induces HIF1α (see, for example, ref. ^45^). Notably, cells of neurovascular units significantly increase *Gal3* messenger RNA with the progression of lesion permeability in CCM animal models^46^.

Since GAL3 is a known activator and regulator of cell proliferation^20^, it may mediate the extra-endothelial proliferation, one of the typical phenotypic characteristics of progressive CCM permeability in patients^46^. GAL3 regulates cell proliferation either by induction of CCND1 expression or by its association with the nuclear mitosis protein NuMa^19,20,22^. Here we show for the first time GAL3 in proliferating human reactive astrocytes entering the G_1_ (as shown by CCND1) and undergoing cell division in regions affected by BBB rupture and hemorrhage. Thus, GAL3 predicts the proliferative status of reactive human astrocytes irrespective of the etiology of the BBB disruption at earlier stages in cell cycle than MIB1(Ki67) and PCNA that label cells in S phase^47^.

### NSC potential elicited by BBB disruption

The adult human cerebral GM is virtually devoid of any detectable neurogenesis in vivo as well as neurosphere formation in vitro, as also shown here. However, in two conditions associated with hemorrhage, namely CCM and epilepsy with IPH, cells from cerebral cortex GM samples formed self-renewing, multipotent neurospheres, highlighting the presence of a small, but reproducibly detected population with NSC hallmarks in vitro. For the total tissue, the frequency of neurosphere formation is in the same range from the endogenous murine NSC niche, the subependymal zone^12^, while GM produced a third to half of the murine stem cell niche’s neurospheres. This highlights the substantial nature of this reaction and provides to our knowledge the first evidence of a population of cells with NSC hallmarks in the adult human cerebral GM. This plastic cell population is an exciting cell source for repair purpose, but also a possible source for the post-TBI glioma formation. For both interventions it would be crucial to know the regulators of this population. GAL3 interacts with numerous signaling cascades involved in stemness, such as the Wnt/β-catenin, Notch, SHH and NF-kB pathways^20,22^ and binds to a number of tyrosine kinase receptors EGFR, TGFR and BMPR1, preventing endocytosis resulting in upregulation of the NSC determinant Sox2 and c-Myc^20,22^. Moreover, we detected a key role of extracellular regulation via the CSF.

### CSF regulates astrocyte proliferation and NSC potential

Importantly, activation of proliferation or neurosphere formation could also be elicited in several in vitro assays from the ICM-affected and even intact human cortical parenchyma upon exposure to the CSF-CCM, but not CSF-ICM. This suggests that the failure of reactive astrocytes to proliferate and mount a NSC response in intact cortex or following ICM-mediated compression is probably due to the lack of environmental signals rather than an intrinsic proliferation block. Indeed, we identified not only diagnosis-specific protein CSF signatures providing novel biomarkers, but also significant enrichment of proteins involved in brain development, neurogenesis and cell proliferation in the CSF-CCM. Interestingly, in both patient groups GAL3 was very low in the CSF, indicating that the pro-proliferative effects of GAL3 on reactive astrocytes takes place most likely intracellularly, like in rodents^21^. Instead, the direct GAL3 interactor LGALS3BP was among the 10% most abundant and significantly enriched CSF-CCM proteins. Despite its implications across many diseases (Supplementary Table 12), most known LGALS3BP functions are related to pathogenesis/progression of several human cancers^48^. However, LGALS3BP is important for human radial glia during neurogenesis and development of human cerebral cortex^49^. Given the similarity between radial glia and proliferating and neurosphere-forming reactive astrocytes^9^, it is intriguing that LGALS3BP acts on both these populations. This is concluded, as the effects of CSF-CCM were prevented by LGALS3BP inhibition with the function-blocking antibody MDP1959. The potential therapeutic benefit of MDP1959-mediated inhibition of LGALS3BP for the treatment of human neuroblastoma was highlighted in different experimental models^48,50^. Therefore, further studies are warranted to assess the efficacy of MDP1959 to reduce uncontrolled astrocyte proliferation triggering malignant transformation in astrocytic brain tumors and the emergence of glioma formation after TBI^7^.

Functionally, LGALS3BP actuates a ‘cell growth’ program in cancer^35,50^ and can induce N-glycosylation intensifying its GAL3 interaction. LGALS3BP binding to GAL3 mediated the Ras/MAPK/ERK cascade promoting cell cycle progression through induction of Ccnd1 and activation of Cdk2/Cdk4 (refs. ^19,26,35,48^). Thus, LGALS3BP can mediate the effects of the CSF-CCM on GAL3^+^ astrocytes promoting their proliferation and neurosphere formation. Furthermore, LGALS3BP interactions with integrins translate environmental mitogenic signals to the cell cycle machinery via AKT, JNK and Raf/ERK cascades^38,48,50^, thus providing a possible mechanistic explanation of how LGALS3BP can activate human astrocyte proliferation and dedifferentiation in the absence of GAL3, such as in ICM cells that upregulate integrins^31^. LGALS3BP may thus act as a functional platform that integrates the environmental cues to activate the proliferative program in reactive human astrocytes.

Taken together, our discovery of in vivo astrocyte proliferation and its molecular hallmarks provides new tools to monitor this response in relation to disease progression. Furthermore, the functional LGALS3BP pathway may act as target to either augment for repair and scar reduction to avoid conditions such as epilepsy development, and/or to reduce its longer-term presence to avoid glioma formation. This work thus paves the way to diagnose and modulate a highly specific astrocyte reaction for intervention with disease progression as well as activate the NSC properties of these cells as an exciting therapeutic entry point for repair and neuronal replacement strategies.

## Methods

### Human tissue and the CSF samples

In this study we used samples of human cerebral cortex tissue obtained during the neurosurgical resection of a single ruptured CCM (*n* = 8), ICM tumor mass (*n* = 4) or of an epileptogenic focus (*n* = 5) (Supplementary Table 1) at Department of Neurosurgery, Clinic of LMU Munich, Germany. Patients were randomly selected according to their diagnosis/clinical state of disease, and each patient over 18 years of age who was surgically treated for CCM, ICM or pharmacologically intractable epilepsy at Department of Neurosurgery (Clinic of LMU Munich) between September 2017 and May 2023 had the same chance of being included in this study. No additional randomization was used during data collection. In accordance to the Declaration of Helsinki and with the ethical guidelines approved by the Ethical Committee at the LMU Munich, Germany (Certificate of Compliance No. 17-263), all patients gave written informed consent to participate in this study, including the collection of samples for research. Patients received no compensation for participation in this study. According to our ethical permit, all research samples were blinded by irreversibly anonymization immediately after collection, and except a neuropathological diagnosis, no individual participant data (including age and sex) are available.

The supratentorial CCM cases were classified as sporadic if patients harbored a solitary lesion on the susceptibility weighted cranial MRI. The extra-axial ICMs were defined by neurological exam followed by contrast-enhanced CT or MRI and classified as benign (non-atypical/non-anaplastic) meningioma. Pharmacologically intractable epilepsy cases were graded according to the International League Against Epilepsy classification systems^51^. Preoperative imaging (computed tomography and/or MRI) in combination with the neuronavigation/intraoperative monitoring/intraoperative ultrasound system allowed to define and delineate the pathology-affected brain tissue from the surrounding nongliotic parenchyma, as well as to rule out the possibility that the research specimens from epilepsy patients included the hippocampus. All tissue samples were collected separately, immediately transferred into ice-cold phenol-free HBSS medium (Invitrogen 14025-050), and to minimize ex vivo alterations rapidly processed for experiments. When the samples were taken for cell culture experiments, we relied on intraoperative classification of tissue state. The histopathological status of each tissue sample was evaluated by GFAP/Iba1 immunolabeling and then arbitrary trichotomized into mild, moderate and severe gliosis. The subarachnoid CSF samples were collected before the neurosurgical tissue resection was started, and afterwards aliquoted in low-protein binding tubes (Sarstedt 72704600) and stored at −80 °C. Samples with relevant blood contamination were excluded from further analysis.

Individual autopsy and biopsy samples from patients with TBI (*n* = 4), stroke (*n* = 3), COVID-19 (*n* = 3) or AC (*n* = 3) were collected and encapsulated for fixation with 4% paraformaldehyde (PFA) according to a standardized protocol. All AC samples were graded into AC IDH-mutant CNS WHO2/3 according to the latest World Health Organization classification of CNS tumors^52^. The collection of specimens and their using for research occurred in accordance to the legal guidelines of Government of Upper Bavaria (BayKrG Art. 27 Abs. 4) and approved from the Ethical committee at the LMU Munich (Certificate of Compliance No. 225/20S, Declaration of No Objection No. 087-13).

### Immunohistochemistry

After fixation in 4% PFA (Carl Roth 0335.4) for 24–36 h at 4 °C, the CCM and ICM tissue samples were cryoprotected in phosphate-buffered saline (PBS; ThermoFisher 14200-083) with 30% sucrose (Carl Roth, 4621.2) for 24 h at 4 °C, and then cut into 20-µm-thick cryosections. The tissue samples from patients with epilepsy were embedded in 4% agarose subsequently after fixation, and then cut into 50-µm-thick vibratome sections. For immunostainings, the serially collected sections were pretreated with blocking solution (2% bovine serum albumin, 0.5% Triton X-100; both Sigma-Aldrich A2153 and T9284) for 45 min at room temperature (RT) and afterwards incubated for 48 h at 4 °C with primary antibodies against CCND1 (1:200; rabbit IgG polyclonal, ThermoFisher RM-904-SO), FN (1:250; rabbit IgG, Sigma-Aldrich F3648), GAL1 (1:200; rat IgG_2B_ monoclonal, R&D MAB1245, clone 201066), GAL3 (1:200; goat IgG polyclonal, R&D AF1197), GFAP (1:400; mouse IgG_1_ monoclonal, Sigma G3893, clone G-A-5), GFAP (1:500; rabbit IgG polyclonal, Dako Z0334), GFAP (1:250; rabbit polyclonal, Sigma G9269), IBA1 (1:500; rabbit polyclonal, Wako 019-19741), LAM1 (1:250; rabbit polyclonal, Bio-Techne NB300-144), LGALS3BP (1:100; IgG; MPD1959, humanized version of the murine SP-2 monoclonal antibody, MediaPharma Italy), MKI67 (1:200; mouse IgG_1_ monoclonal, Dako M7240 clone MIB1) diluted in PBS. After washing with PBS (3× 10 min, RT), sections were incubated for 4 h at RT with species- and subclass-specific secondary antibodies: anti-mouse IgG Alexa Fluor 488 (1:1,000; Invitrogen A21202), anti-mouse IgG Alexa Fluor 594 (1:1,000; Invitrogen A21203), anti-mouse IgG Alexa Fluor 647 (1:1,000; Invitrogen A32787), anti-human IgG Alexa Fluor 647 (1:500; ThermoFisher 21445), anti-mouse IgM CY3-conjugated (1:1,000; Jackson Immuno Research 715-165-020), anti-goat IgG Alexa Fluor 488 (1:1,000; Invitrogen A11055), anti-goat IgG CY3-conjugated (1:1,000; Jackson Immuno Research 05-165-147) anti-rat IgG Alexa Fluor 488 (1:1,000; Invitrogen A21208), anti-rabbit IgG Alexa Fluor 488 (1:1,000; Invitrogen A31573) and anti-rabbit IgG CY3-conjugated (1:1,000; Jackson Immuno Research 711-165-152) diluted in PBS. For nuclei labeling, sections were incubated with 4’,6-diamidino-2-phenylindole (DAPI; 0.1 mg ml^−1^; Sigma D9542) and mounted on glass slides with Aqua-Polymount (Polysciences 18606) and examined either at an epifluorescence microscope and/or scanned at the confocal microscope to produce digital images.

Paraffin-embedded tissue samples from TBI, stroke or COVID-19 victims or individual biopsy material of AC were cut into 14-µm-thick sections, and after deparaffinization, sections were treated with citrate buffer (10 mM, pH 6, Carl Roth 6779.1) for 15 min at 800 W and then 10 min at 400 W. After washing in PBS, peroxidase activity was quenched in blocking solution (10% H_2_O_2_, Carl Roth 8070.4) in PBS for 30 min RT, and after 3× washing in PBS, sections were incubated overnight at 4 °C with antibody against GAL3 diluted in blocking buffer (10% normal horse serum, Invitrogen 31874; in PBS). After washing in PBS and detection of primary antibody using the 3,3′-diaminobenzidine (DAB Liquid Substrate System tetrahydrochloride, Sigma-Aldrich D7304) sections were counterstained with hematoxylin. For immunofluorescence labeling, consecutive paraffin sections were pretreated with blocking solution (2% bovine serum albumin, 0.5% Triton X-100) for 45 min at RT before incubation with primary and secondary antibodies and DAPI. Sections were then mounted on glass slides and scanned at the confocal microscope to produce digital images.

### hiPS cell-derived astrocytes

hiAstros were differentiated from three iPS cell lines: #1 (known as ISFi001-A/HMGU1) and HMGU12 (known as ISFi0012-A) generated and readily available from the iPS cell core unit at the Helmholtz Zentrum München; UKERi82a-R1-002 (known as ERF31E2)^53–55^ obtained as part of the ForIPS^56^ research consortium (Ethics Approval No. 4120, FAU Erlangen-Nuernberg, Germany)and not available for third parties (see https://hpscreg.eu/cell-line/UKERi006-A). For detailed genetic information, see https://hpscreg.eu. hiAstros were generated as previously described^57^ with some alterations. Briefly, confluent iPS cell cultures were cultured 3 weeks in suspension to form embryoid bodies. The cells were cultured in mTESR1 with 1× mTESR1 supplement and 10 μM Rock Inhibitor Y-27632 (both STEMCELL Technologies 85850 and 72304) for the first 24 h, and then for the next 2 weeks in astrocyte medium (AM; ScienCell 1801) supplemented with 20 ng ml^−1^ Noggin and 10 ng ml^−1^ PDGFAA (both Peprotech 120-10C9, 100-13A). In the 3 weeks of suspension, the cells were only treated with PDGFAA. Embryoid bodies were then manually dissociated by pipetting and plated for differentiation in AM supplemented with fibroblast growth factor 2 (FGF2) and epidermal growth factor (EGF) (both at 10 ng ml^−1^ medium; Peprotech 100-18B, 100-15). The human induced glial progenitor cells (hiGPCs) were cultured on poly-l-ornithine (PO)- and laminin- (both Sigma-Aldrich P3655, L2020) coated dishes and were passaged with Accutase (Sigma-Aldrich A6964) once the cells reached ~80% confluency. After 46 days of differentiation, astrocyte maturation was induced by AM supplemented with 10 ng ml^−1^ LIF (Peprotech 300-05). The cells were collected on day 60 of differentiation and used for immunocytochemistry with S100B (1:250; mouse IgG_1_ monoclonal, Sigma S2532, clone SH-B1) and FGFR3 (1:50; rabbit polyclonal, Santa Cruz sc-123) antibodies.

For proliferation assay, hiAstros at day 60 were plated on the PO/laminin-coated eight-chamber slides (ThGeyer 62001671) (25,000 cells per well) in 200 μl LIF-supplemented AM containing EdU (10 μM ml^−1^; Life Technologies E10187). CSF-CCM or CSF-ICM (100 μl ml^−1^ medium), MDP1959 (40 μl ml^−1^ medium) and/or recombinant human (rh) LGALS3BP (10 μg ml^−1^ or 20 μg ml^−1^ medium; R&D Systems 2226-GAB) were added at the beginning of the experiment only. After 7 days, cells were fixed in 4% PFA, washed with PBS (3× 10 min, RT) and then incubated with primary antibodies against S100B (1:250; mouse IgG_1_ monoclonal, Sigma, S2532, clone SH-B), GFAP (1:250; mouse IgG_1_ monoclonal, Sigma G3893), GAL3 (1:100) or LGALS3BP (1:100) for 30 min at RT. After washing in PBS (3× 10 min, RT), cells were incubated with species- and subclass-specific secondary antibodies for 30 min at RT. EdU detection was performed with the Click-iT EdU Imaging Kit (according to the manufacturer’s instruction, ThermoFisher C10340). For nuclear labeling, cells were incubated with DAPI for 15 min at RT and mounted on glass slides.

### Neurosphere cultures

Neurosphere cultures were prepared with cells dissociated from the tissue samples as well as with hiAstros at day 60. Briefly, after enzymatic dissociation with 0.025% trypsin (Gibco 25200-056) (15 min, 37 °C), cells were plated into single wells (24-well plates, Sarstedt 83.3922.005) at a density of one to five cells per milliliter in neurosphere medium (Dulbecco’s modified Eagle medium/F12, Gibco 21331-046; with 1% vol/vol PenStrep, 1% vol/vol B27 and 2 mM l-glutamine, all Invitrogen 15140-122, 17504044 and 25030081) supplemented with FGF2 and EGF (both at 20 ng ml^−1^ medium) per well. In five independent experiments, GM and WM were separated before the dissociation of tissue samples from four patients with CCM, and cells derived from GM and WM of intact, mild and moderate gliosis regions were used. After 7 d.i.v. at 37 °C and 5% CO_2_, half of the neurosphere medium was replaced with fresh growth factors-containing medium. In the hiAstros-derived neurosphere cultures, 50 μl of fresh growth factors-containing medium (both at 40 ng ml^−1^) was added at 7 days after plating. In some experimental sets, CSF from patients with CCM or ICM (100 μl ml^−1^ medium) and/or MDP1959 (40 μl ml^−1^ medium) and/or rhLGALS3BP (10 μg ml^−1^ or 20 μg ml^−1^ medium) were added only at the beginning of the experiment. The working concentration of rhLGAL3BP was chosen on the basis of previously reported dose–response experiments^58,59^.

After counting on day 14, primary neurospheres were assessed for capacity to self-renew, as previously described^21,60^. In short, neurospheres were incubated with 0.125% trypsin (15 min, 37 °C) and afterwards mechanically dissociated into a single-cell suspension and centrifuged. Then cells were re-suspended with neurosphere medium and plated for 14 days in three to six wells at the same density during the primary culture. The number of neurospheres in each cell culture condition was quantified after 14 d.i.v., and the incidence of neurosphere-forming cells per 10,000 cells was calculated. Nonspherical cell clusters as well as neurospheres smaller than 50 μm in diameter were excluded from counts. For differentiation assay, three to five individual neurospheres (≥50 μm in diameter) per cell culture condition were plated onto PO/laminin-coated in growth factor-free neurosphere medium containing 1% fetal calf serum (PAN Biotech P30-3302). After 7 days, the type of differentiated cells in the migration area was assessed by triple immunolabeling for βIII-tubulin (1:300; guinea pig polyclonal, SynapticSystems 302 304), GFAP (1:1,000; rabbit polyclonal, Dako Z0334) and O4 (1:50; mouse IgM monoclonal, Sigma O7139 clone O4).

### Image acquisition

All confocal images were acquired with a Zeiss LSM710 (Carl Zeiss) microscope using the ZEN software (black edition, v.2.3 SP1). Fluorescence imaging of immunolabeled cell cultures was performed with an epifluorescence microscope Zeiss Axio Imager M2 (Carl Zeiss) using the ZEN 2pro software. All phase-contrast images were acquired with Leica DMIL LED (Leica) using Leica Application Suite-LAS software (v.4.6).

### Proteome analysis of the CSF

Total protein content of CSF samples was measured by Bradford assay (Bio-Rad) and 10 µg per sample were proteolysed by the commercially available in-StageTip-NHS kit (PreOmics GmbH) according to the manufacturer’s protocol. Briefly, CSF was reduced and alkylated and incubated for 3 h at 37 °C with Lys-C and trypsin. Resulting peptides were dried for short-term storage at −80 °C. Before measurement, peptides were resuspended in 2% acetonitrile and 0.5% trifluoroacetic acid. Peptides were analyzed on a Q Exactive HF mass spectrometer (Thermo Fisher Scientific) online coupled to a UItimate 3000 RSLC nano-HPLC (Dionex). Samples were automatically injected and loaded onto the C18 trap cartridge and after 5 min eluted and separated on the C18 analytical column (Acquity UPLC M-Class HSS T3 Column, 1.8 μm, 75 μm × 250 mm; Waters) by a 90 min nonlinear acetonitrile gradient at a flow rate of 250 nl min^−1^. MS spectra were recorded at a resolution of 60,000 with an automatic gain control (AGC) target of 3 × 10^6^ and a maximum injection time of 30 ms from 300 to 1,500 *m*/*z*. From the MS scan, the ten most abundant peptide ions were selected for fragmentation via HCD (higher collisional dissociation) with a normalized collision energy of 27, an isolation window of 1.6 *m*/*z*, and a dynamic exclusion of 30 s. MS/MS spectra were recorded at a resolution of 15,000 with an AGC target of 1 × 10^5^ and a maximum injection time of 50 ms. Unassigned charges, and charges of +1 and >8 were excluded from precursor selection.

Acquired raw data were analyzed in the Proteome Discoverer 2.4 SP1 software (Thermo Fisher Scientific; version 2.4.1.15) for peptide and protein identification via a database search (Sequest HT search engine) against the SwissProt Human database (Release 2020_02, 20432 sequences), considering full tryptic specificity, allowing for up to one missed tryptic cleavage site, precursor mass tolerance 10 ppm, fragment mass tolerance 0.02 Da. Carbamidomethylation of cysteine was set as a static modification. Dynamic modifications included deamidation of asparagine and glutamine, oxidation of methionine, and a combination of methionine loss with acetylation on protein N-terminus. The Percolator algorithm^61^ was used for validating peptide spectrum matches and peptides. Only top-scoring identifications for each spectrum were accepted, additionally satisfying a false discovery rate (FDR) <1% (high confidence). The final list of proteins satisfying the strict parsimony principle included only protein groups passing an additional protein confidence FDR <1% (target/decoy concatenated search validation). Quantification of proteins, after precursor recalibration, was based on abundance values (area under the curve) for the unique peptides per protein. Peptide abundance values were normalized on total peptide amount. The protein abundances were calculated summing the abundance values for admissible peptides. The final protein ratio was calculated using median abundance values of the biological replicates in a nonnested design. The MS proteomics data have been deposited to the ProteomeXchange Consortium via the PRIDE partner repository with the dataset identifier PXD045579.

The identified 860 proteins with ≥2 unique peptides and FDR <1% were run against the existing human proteomics datasets of the neural tissue from different CNS regions, CSF and peripheral blood using the webserver ProteomicsDB^62^ (https://www.proteomicsdb.org/). Heatmaps in Extended Data Fig. 4b–e were generated with the ProteomicsDB database analytic toolbox expression heatmap (by selecting MS1, Tissue, Fluid and Swissprot only). Heatmap of the 28 proteins that were exclusively detected in all CSF-CCM samples was generated with GraphPad Prism (v10) and is depicted in Fig. 4b. Predicted disease associations for the top 45 most abundant CSF-CCM proteins were analyzed using GS2D^63^ database^[]^.

### GO enrichment analysis and protein interaction networks

GO, KEGG and REACTOM pathway enrichment analyses were performed for significantly differentially expressed up- or downregulated proteins using the STRING^64^ database v.11 (https://string-db.org/). A protein network analysis was performed using the STRING database of known PPI, with the following settings: interaction sources were experiments and databases, and minimum required interaction score was set to medium confidence. Proteins were connected on the basis of confidence, and disconnected nodes were discarded. Resulting PPIs for 483 proteins expressed at comparable levels across all CSF samples (5,228 edges, *P* < 1 × 10^−16^), for 62 proteins enriched in CSF-ICM (262 edges, *P* < 1 × 10^−16^) and for the 158 most enriched CSF-CCM proteins (497 edges, *P* < 1 × 10^−16^) are shown in Extended Data Figs. 5d, 5h and 6a, respectively. PPI network of experimentally validated LGALS3BP interactions (Fig. 4j) was generated with confidence score >400 using the STRING^64^ database. The prediction of the possible gene association network and functions of the top 45 most abundant significantly upregulated proteins defining the CSF-CCM signature was performed using an integrated interaction network program GeneMANIA Human Database^65^ (Gene Function Prediction using a Multiple Association Network Integration Algorithm; 3.6.0, www.genemania.org), and the resulting networks are given in Fig. 4h and Extended Data Fig. 6b–e.

### Statistics and reproducibility

All experimental groups were composed of at least three biological replicates (*n*) identified by diagnosis/histopathological state of tissue samples from patients and cell culture condition, or iPS cell line and culture condition. Sample sizes were selected on the basis of previous reports^17^^,21,66^. All experiments were performed at least three times independently, and results were reproducible. Quantifications and colocalization analysis were performed blindly and in a minimum three sections per tissue sample per patient and in all individual *z*-planes (spaced at 1.5 μm) across serial optical *z*-stack of a minimum of three nonoverlapping regions per section or for cell culture experiment in at least three biological replicates per culture condition and by counting on average 200 cells per cell culture within a minimum of three randomly selected nonoverlapping fields. Percentage of immunolabeled cells in each culture condition was calculated per biological replicate per iPS cell line. Group sizes are defined by individual data points in each plot and are clearly stated in figure legends. Micrographs shown in figures are representative for *n* ≥ 3 biological replicates: Figs. 1d–f (*n* = 8), 2a–e,g–k (*n* = 4), 3a–f (*n* = 5), 5b,g (*n* = 5), 5i (*n* = 3) and 6f–l (*n* = 4). No data were excluded from the analysis, and no samples were measured repeatedly.

Unless stated otherwise, all statistical data analyses were performed with Prism 10 software (v.10.0.0 (131) (GraphPad). Normality of data distribution was tested using the Shapiro–Wilk test, and statistical significance of normally distributed data was tested with unpaired *t*-test with Welch’s correction for two-group comparison, or ordinary one-way analysis of variance (ANOVA) followed by Tukey’s or Holm–Šídák’s multiple comparisons test, Brown–Forsythe and Welch ANOVA for more than two groups and in the case of significantly different standard deviations within data groups. For nonnormally distributed data, statistical significance for two-group comparison was tested with Mann–Whitney, and Kruskal–Wallis test and Dunn’s for multiple comparisons were used for more than two groups. Statistical significance of the protein fold change was ascertained with Bonferroni-corrected Welch *t*-test of log transformed data with imputed values equal 0.0001 selecting for proteins with at least five replicates with nonzero values in CSF-CCM. Differential protein expression analysis using FC ≥2 and adjusted *P*‐value ≤ 0.05 was performed using R statistical framework (R Foundation for Statistical Computing, http://www.R-project.org/) and matrixTests package (version 0.1.9). All statistical tests and post-hoc tests are indicated in the figure and tables legends.

### Reporting summary

Further information on research design is available in the Nature Portfolio Reporting Summary linked to this article.

## Online content

Any methods, additional references, Nature Portfolio reporting summaries, source data, extended data, supplementary information, acknowledgements, peer review information; details of author contributions and competing interests; and statements of data and code availability are available at 10.1038/s41591-023-02644-6.

### Supplementary information

Reporting Summary

Supplementary Tables **Supplementary Table 1**. Characteristics of surgically resected specimens. **Supplementary Table 2**. List of 860 proteins (≥2 unique peptides and FDR <0.01) identified in CSF samples from ICM and CCM patients. **Supplementary Table 3**. List of nonregulated proteins (*P* > 0.05 and/or FC <2 or >−2) in patient CSF samples. **Supplementary Table 4**. List of significantly upregulated proteins (*P* ≤ 0.05 and FC ≥ 2) in CSF-CCM versus CSF-ICM. **Supplementary Table 5**. List of significantly downregulated proteins (*P* ≤ 0.05 and FC ≤ −2) in CSF-CCM versus CSF-ICM. **Supplementary Table 6**. Significantly enriched biological process GO terms related to the upregulated CSF-CCM proteins (*P* ≤ 0.05 and FC ≥ 2). **Supplementary Table 7**. The top five significantly enriched REACTOME pathways related to the downregulated CSF-CCM proteins (*P* ≤ 0.05 and FC ≤−2). **Supplementary Table 8**. The top five significantly enriched REACTOME pathways related to the upregulated CSF-CCM proteins (*P* ≤ 0.05 and FC ≥2). **Supplementary Table 9**. List of the top 158 significantly up-regulated proteins (*P* ≤ 0.05 and FC ≥ 5) in CSF-CCM versus CSF-ICM. **Supplementary Table 10**. List of the top 45 most abundant significantly enriched (*P* ≤ 0.05 and FC ≥ 3) proteins defining signature of CSF-CCM. **Supplementary Table 11**. The cellular components identified by GO enrichment analysis for the top 45 most abundant proteins (*P* ≤ 0.05 and FC ≥ 3) defining signature of CSF-CCM. **Supplementary Table 12**. GS2D predicted disease associations for the top 45 most abundant and significantly upregulated CSF-CCM proteins (*P* ≤ 0.05 and FC ≥ 3)

### Source data

Source Data Fig. 2 Statistical source data.

Source Data Fig. 3 Statistical source data.

Source Data Fig. 4 Statistical source data.

Source Data Fig. 5 Statistical source data.

Source Data Fig. 6 Statistical source data.

Source Data Extended Data Fig. Statistical source data.

Source Data Extended Data Fig. Statistical source data.

Source Data Extended Data Fig. Statistical source data.

Source Data Extended Data Fig. Statistical source data.

Source Data Extended Data Fig. Statistical source data.

## Data Availability

The data that contribute to the findings of this study are available within the article and included in supplementary files. The MS proteomics data have been deposited to the ProteomeXchange Consortium via the PRIDE partner repository with the dataset identifier PXD045579. Further data supporting the findings of this study are available from the corresponding authors upon reasonable request. Restrictions apply to the availability of individual participant data. Source data are provided with this paper.
