# Supplementary material for: Injury-specific factors in the cerebrospinal fluid regulate astrocyte plasticity in the human brain
Source: Nat Med. 2023 Dec 8;29(12):3149–61. doi: 10.1038/s41591-023-02644-6 (PMC10719094; doi:10.1038/s41591-023-02644-6)
Supplement: Supplementary file 1 — Reporting Summary [file 41591_2023_2644_MOESM1_ESM.pdf]

nature portfolio

NMED-A122136B
Corresponding author(s): Magdalena Götz and Swetlana Sirko
Last updated by author(s): Oct 11, 2023

Reporting Summary

Nature Portfolio wishes to improve the reproducibility of the work that we publish. This form provides structure for consistency and transparency in reporting. For further information on Nature Portfolio policies, see our Editorial Policies and the Editorial Policy Checklist.

Statistics

For all statistical analyses, confirm that the following items are present in the figure legend, table legend, main text, or Methods section.

n/a	Confirmed	
☐	☒	The exact sample size (*n*) for each experimental group/condition, given as a discrete number and unit of measurement
☐	☒	A statement on whether measurements were taken from distinct samples or whether the same sample was measured repeatedly
☐	☒	The statistical test(s) used AND whether they are one- or two-sided<br>*Only common tests should be described solely by name; describe more complex techniques in the Methods section.*
☒	☐	A description of all covariates tested
☐	☒	A description of any assumptions or corrections, such as tests of normality and adjustment for multiple comparisons
☐	☒	A full description of the statistical parameters including central tendency (e.g. means) or other basic estimates (e.g. regression coefficient) AND variation (e.g. standard deviation) or associated estimates of uncertainty (e.g. confidence intervals)
☐	☒	For null hypothesis testing, the test statistic (e.g. *F*, *t*, *r*) with confidence intervals, effect sizes, degrees of freedom and *P* value noted<br>*Give P values as exact values whenever suitable.*
☒	☐	For Bayesian analysis, information on the choice of priors and Markov chain Monte Carlo settings
☒	☐	For hierarchical and complex designs, identification of the appropriate level for tests and full reporting of outcomes
☒	☐	Estimates of effect sizes (e.g. Cohen's *d*, Pearson's *r*), indicating how they were calculated

Our web collection on statistics for biologists contains articles on many of the points above.

Software and code

Policy information about availability of computer code

Data collection	Zeiss LSM710 (Carl Zeiss) laser-scanning confocal microscope<br>Zeiss Axio Imager M2 (Carl Zeiss) epifluorescence microscope<br>Leica DMIL LED (Leica)
Data analysis	Digital images: Confocal and epifluorescence images were acquired and analyzed with the ZEN (black edition, v.2.3 SP1) software and ZEN 2pro software (Zeiss; https://www.zeiss.com/microscopy/en_us/poducts/microscope-software/zen.html RRID:SCR_013672). Phase-contrast images of neurosphere cultures were obtained with Leica DMIL LED microscope and documented with LAS software (V4.6).<br>Label-free quantitative LC-MS/MS analysis of CSF samples: Q Exactive HF mass spectrometer (Thermo Fisher Scientific) online coupled to a Ultimate 3000 RSLC nano-HPLC (Dionex) was used to analyze peptides. Acquired raw data was analyzed in the Proteome Discoverer 2.4 SP1 software (Thermo Fisher Scientific; version 2.4.1.15) for peptide and protein identification via a database search (Sequest HT search engine) against the SwissProt Human database (Release 2020_02, 20432 sequences). The Percolator algorithm (Kall et al. 2007; PMID 17952086) was used for validating peptide spectrum matches and peptides. R statistical framework (R Foundation for Statistical Computing, Vienna, Austria http://www.R-project.org/) (open source) was used for statistical analysis of the datasets.<br>Gene Ontology (GO) enrichment analysis on Biological Process (BP), Cellular Compartment (CC) or Molecular Function (MF), KEGG, REACTOM pathways and network analysis were performed using open sources STRING database v.11 (https://string-db.org/) and GeneMANIA (Gene Function Prediction using a Multiple Association Network Integration Algorithm; 3.6.0; www.genemania.org). Disease-association analysis of the enriched CSF proteins was performed using Gene Set to Diseases (GS2D) database (http://cbdm-01.zdv.uni-mainz.de/) (open source) and comparission on webserver ProteomicsDB (https://www.proteomicsdb.org/). The identified 860 proteins with ≥2 unique peptides and FDR < 1% were run against the existing human proteomics datasets of the neural tissue from different CNS regions as well as 'pooled footprint' of CSF and peripheral blood using the webserver ProteomicsDB (https://www.proteomicsdb.org/) and an expression heatmaps generated from ProteomicsDB are given in Extended Data Figure 4.

Statistical analysis of acquired data in this study was performed using GraphPad Prism software (v.10) http://www.graphpad.com RRID:SCR_002798) and Microsoft Excel (http://www.graphpad.com RRID:SCR_002798).
All figures in the paper were prepared using Adobe Illustrator (https://www.adobe.com RRID:SCR_010279).

For manuscripts utilizing custom algorithms or software that are central to the research but not yet described in published literature, software must be made available to editors and reviewers. We strongly encourage code deposition in a community repository (e.g. GitHub). See the Nature Portfolio guidelines for submitting code & software for further information.

Data

Policy information about availability of data

All manuscripts must include a data availability statement. This statement should provide the following information, where applicable:

- Accession codes, unique identifiers, or web links for publicly available datasets
- A description of any restrictions on data availability
- For clinical datasets or third party data, please ensure that the statement adheres to our policy

The data that contribute to the findings of this study are available within the article and included in supplementary files. The mass spectrometry proteomics data have been deposited to the ProteomeXchange Consortium via the PRIDE partner repository with the dataset identifier PXD045579.
Further data supporting the findings of this study are available from the corresponding authors upon reasonable request. Restrictions apply to the availability of individual participant data. Source data are provided with this paper.

Human research participants

Policy information about studies involving human research participants and Sex and Gender in Research.

Reporting on sex and gender	Sex and gender were not considered in study design. According to our ethical permit, the biomaterials were made available for research purposes only after irreversible anonymization immediately after collection, so we have no information regarding sex and gender of the patients.
Population characteristics	The samples used in this study were obtained from patients over 18 years of age. We have no information regarding population characteristics, as per our ethical permit,only a neuropathological diagnosis and a minimum subject age are available participant data. The diagnosis, clinical features and status of neurological disorder were defined and/or confirmed by neurologists at the Department of Neurosurgery, Clinic of Ludwig-Maximilians University Munich, Germany. According to the currently accepted disease categorization, the supratentorial CCM cases were classified as sporadic if patients harbored a solitary lesion on the susceptibility weighted cranial MR imaging. The type of extraaxial tumor was defined as ICM thorough neurological exam followed by an imaging with contrast-enhanced CT or MR imaging and classified as benign (non-atypical/ non-anaplastic) meningioma. Cortical dysplasia (FCD) and/or mesial temporal sclerosis (MTS) in patients operated for pharmacologically intractable epilepsy were graded according to the International League Against Epilepsy classification systems. Astrocytoma specimens were examined microscopically and graded into Astrocytoma IDH-mutant CNS WHO grade 2/3 according to the latest WHO classification of CNS tumors.
Recruitment	Patients undergoing neurosurgery at at the Department of Neurosurgery, Clinic of Ludwig-Maximilians University Munich (Germany) between September 2017 and May 2023 were eligible to participate if they were at least 18 years of age and consented to donate tissue during their surgery. Participants were recruited during the course of a pre-operative explanatory meeting in the Department of Neurosurgery (Clinic of Ludwig-Maximilians University Munich, Germany) and only patients who gave the written informed consent to participate in this study were allocated to the one of two diagnosis-related patient groups. Neither the indication for surgery, the surgical technique/strategy or the overall therapeutic concept were influenced by this.<br>Individual autopsy and biopsy samples were collected according to a standardized protocol and were occured after completion of examination at the Institute of Forensic Medicine (Ludwig-Maximilians University in Munich, Germany) and according to the following inclusion criteria: (i) the order of a forensic autopsy by the local prosecutor, (ii) older than 18 years of age, (ii) an acute phase after TBI and (iv) the minimal autolytic changes of the brain tissue.
Ethics oversight	This study was approved by the Ethical committee at Ludwig-Maximilian-University Munich, Germany (Ethics Approval no. 17-263). The collection of individual autopsy and biopsy specimens and their using for research occurred in accordance to the legal guidelines of Government of Upper Bavaria (BayKrG Art. 27 Abs. 4) and approved from the Ethical committee at the LMU Munich (Certificate of Compliance No. 225/20S, Declaration of no objection No. 087-13).

Note that full information on the approval of the study protocol must also be provided in the manuscript.

Field-specific reporting

Please select the one below that is the best fit for your research. If you are not sure, read the appropriate sections before making your selection.

☒ Life sciences ☐ Behavioural & social sciences ☐ Ecological, evolutionary & environmental sciences

For a reference copy of the document with all sections, see nature.com/documents/nr-reporting-summary-flat.pdf

Life sciences study design

All studies must disclose on these points even when the disclosure is negative.

Sample size	No statistical methods were used to pre-determine size of samples. Sample sizes were selected based on previous reports for similar experiments and were deemed sufficient to perform an non-clinical study.
Data exclusions	Two CSF samples (one from CCM group of patients and one from ICM group of patients) were excluded from the analysis due to high levels of blood contamination.
Replication	All experiments were repeated at least three times with and the results could be replicated each time. The number of replicates runs for each experiment and sample size are provided in the main text, figure legends and the method section.
Randomization	Biospecimens from patients were grouped based on the neurological diagnosis and independent from data collection. Throughout the whole experiments, all specimens were taken into account and analyzed equally, so there was no requirement for randomization.<br>All experiments with human astrocytes derived from three hIPSC lines were done without any pre-selection or randomization. Different batches of frozen hIPSC-derived glial progenitor cells were selected randomly. On day 60 of astroglial differentiation, cells were collected from a coated dishes and split into different cell culture conditions. No additional randomization was used during data collection.
Blinding	All stereotactic obtained neurosurgical tissue samples were classified into regions (e.g. 1-4) according to the results of both preoperative imaging (ie. CT and/or MRI (T2 weighted) and intraoperative monitoring (IONM), incl. intraoperative ultrasound (IOUS). Consistent with conventional diagnostic evaluation, histopathological status/severity of reactive gliosis of all stereotactic obtained specimens was classified and graded in an arbitrary 3 tier scale with the help of GFAP/Iba1 immunolabeling. When tissue was taken for in vitro experiments, we relied on the intraoperative region classification, e.g. 1-4, and thus in a blinded fashion. Investigators were also blinded during analysis of all individual autopsy and biopsy samples, as well as to the cell culture conditions with human hiPSC-derived astrocytes.<br>The quantitative label-free LC-MS/MS analysis of CSF samples was done by researchers blinded to patient diagnosis and the results of tissue analysis, i.e., the researchers during data collection and initial analysis were not aware of the sample group allocation. Once this part of analysis was completed, the group allocation was revealed for the Gene Ontology enrichment analysis and generation of protein interaction networks.

Reporting for specific materials, systems and methods

We require information from authors about some types of materials, experimental systems and methods used in many studies. Here, indicate whether each material, system or method listed is relevant to your study. If you are not sure if a list item applies to your research, read the appropriate section before selecting a response.

Materials & experimental systems

n/a	Involved in the study	
☐	☒	Antibodies
☐	☒	Eukaryotic cell lines
☒	☐	Palaeontology and archaeology
☒	☐	Animals and other organisms
☒	☐	Clinical data
☒	☐	Dual use research of concern

Methods

n/a	Involved in the study	
☒	☐	ChIP-seq
☒	☐	Flow cytometry
☒	☐	MRI-based neuroimaging

Antibodies

Antibodies used

All antibodies used in this study are all listed in the Methods section.
Primary antibodies:
1) anti-βIII-tubulin (1:250; mouse IgG2b monoclonal, Sigma, cat. no T8660 sc-5274, lot no 046M4819V)
2) anti-βIII-tubulin (1:300; guinea pig polyclonal, SynapticSystems, cat. no 320304, lot no.1-19a)
3) anti-CCND1(1:200; rabbit IgG polyclonal, Thermo Fisher cat. no. RM-904-SO, lot no. 9104S1308C)
4) anti-FGFR3 (1:50; rabbit IgG polyclonal, Santa Cruz, cat. no. sc-123, lot no. L1808)
5) anti-FN (1:250; rabbit IgG, Sigma-Aldrich cat. no. F3648, lot no. 086K4803)
6) anti-GAL1(1:200; rat IgG2B monoclonal, clone 201066, R&D cat. no. MAB1245, lot no. JGQ0211071)
7) anti-GAL3 (1:200; goat IgG polyclonal, R&D cat. no. AF1197, lot no. JAA0111081)
8) anti-GFAP (1:400; mouse IgG1 monoclonal, clone G-A-5, Sigma-Aldrich cat. no. G3893, lot no. 119M4802V)
9) anti-GFAP (1:500; rabbit IgG polyclonal, Dako cat. no. Z0334, lot no. 20069186, 167864 and 41387723)
10) anti-GFAP (1:250; rabbit IgG polyclonal, Sigma-Aldrich cat. no. G9269, lot no.0000193774)
11) anti-IBA1 (1:500; rabbit IgG polyclonal, Wako cat. no. 019-19741, lot no. SKP3626)
12) anti-MKI67 (1:200; mouse IgG1 monoclonal, clone MIB1, Dako cat. no. M7240, lot no.20069186 and 20023412),
13) anti-LAM1 (1:250; rabbit IgG polyclonal, Bio-Techne cat. no. NB300-144, lot no. 2923-071822)
14) anti-LGALS3BP/MDP-1959 (1:100; IgG; MPD1959, humanized version of the murine SP-2 monoclonal antibody, MediaPharma Italy)
15) anti-O4 (1:50; mouse IgM monoclonal, clone O4, Sigma, cat. no. O7139, lot no. SLBX6296)
16) anti-S100B (1:250; mouse IgG1 monoclonal, clone SH-B1, Sigma, cat. no. S2532, lot no. 131545)

	Secondary antibodies:<br>1) anti-goat IgG Alexa Fluor 488 (1:1000; Invitrogen cat. no. A11055, lot no. 2411589)<br>2) anti-goat IgG CY3-conjugated (1:1000; Jackson Immuno Research cat. no. 05-165-147, lot no. 154585)<br>3) anti-human IgG Alexa Fluor 647 (1:500; Thermo Fisher cat. no. 21445, lot no. 2090672)<br>4) anti-mouse IgG Alexa Fluor 488 (1:1000; Invitrogen cat. no. A21202, lot no. 2266877)<br>5) anti-mouse IgG Alexa Fluor 594 (1:1000; Invitrogen cat. no. A21203, lot no. 2134005)<br>6) anti-mouse IgG Alexa Fluor 647 (1:1000; Invitrogen cat. no. A32787, lot no. VJ307587)<br>7) anti-mouse IgM CY3-conjugated (1:1000; Jackson Immuno Research cat. no. 715-165-020, lot no. 157626)<br>8) anti-rabbit IgG Alexa Fluor 488 (1:1000; Invitrogen cat. no. A31573, lot no. 1981125)<br>9) anti-rabbit IgG CY3-conjugated (1:1000; Jackson Immuno Research cat. no. 711-165-152, lot no. 15599510)<br>10) anti-rat IgG Alexa Fluor 488 (1:1000; Invitrogen cat. no. A21208, lot no.2273677)
Validation	1) anti-βIII-tubulin has been validated in multi-lineage differentiated human stem cells (Zhang et al. 2016, PMID: 27151462)<br>2) anti-βIII-tubulin has been validated in hiPSC-derived cortical neurons (Xie et al. 2023, PMID: 37423307)<br>3) anti-CCND1 has been validated in human cells (Logan et al. 2012, PMID: 22534328)<br>4) anti-FGFR3 has been validated in human cells and tissue samples (Vlacic et al. 2019, PMID: 31527449)<br>5) anti-FN has been validated in human clear cell carcinom (An et al. 2023, 37009792)<br>6) anti-GAL1 has been validated in mouse brain tissue (Plachta et al. 2007, PMID: 17486104) and in human brain tissue by investigators, using secondary antibody controls (withdrawal of primary antibody) and by localization and morphology of the detected cells<br>7) anti-GAL3 has been validated in mouse brain tissue (Sirko et al. 2015, PMID: 17486104), human tissue (Buhimschi et al. 2009, PMID: 19679874) and human iPSC-derived astrocytes by investigators, using secondary antibody controls (withdrawal of primary antibody) and by detected localization in the cells and their environment<br>8) anti-GFAP has been validated in human brain tumors (Weng et al. 2019, PMID: 30982771) and in human iPSC-derived astrocytes (Li et al. 2018, PMID: 30075130)<br>9) anti-GFAP has been validated previously in human brain tissue (Leonard et al. 2009, PMID: 19425077)<br>10) anti-GFAP has been validated previously in human brain tumours (Saadoun et al. 2002, PMID: 11796780) and human brain cells in patients with Alexander disease (Yoshida et al. 2013, PMID: 23903069)<br>11) anti-IBA1 has been validated in human brain tissue and glioma cell lines (Kuan et al. 2016, PMID: 27632900; Dekens et al. 2017, PMID: 27716662, Keane et al., 2021, PMID: 34485907)<br>12) anti-MIK67/clone MIB1 has been validated in human tissue (Gerdes et al. 1992, PMID: 1453271), including human brain and neural tumors (Katyal et al. 2011, PMID: 22163036, Popova et al. 2014, PMID: 24410805)<br>13) anti LAM1 has been validated in different types of human tissie and cells (Ashok et al. 2019, PMID: 31511544)<br>14) anti-LGALS3BP/MDP-1959 has been validated previously in human cell lines and blood plasma (Giansanti et al. 2019, PMID: 30553852; Capone et al. 2020, PMID: 33076448; Gallo et al. 2022, PMID: 35076790) as well as in human iPSC-iderived astrocytes by investigators, using secondary antibody controls (withdrawal of primary antibody) and by cellular localization of the detected structures<br>15) anti-O4 has been validated in human iPSC-derived oligodendrocyte progenitor cells (Wang et al. 2013, PMID: 23395447)<br>16) anti-S100B has been validated in human iPSC-derived astrocytes (Sareen et al. 2014, PMID: 24610630), human serum (Duda et al. 2017, PMID: 29222857) and himan brain tissue (Almad et al. 2016, PMID: 27083773).<br><br>For more information about validation procedures of all antibodies used in this study see the sites of the companies/providers.

Eukaryotic cell lines

Policy information about cell lines and Sex and Gender in Research

Cell line source(s)	For this study, human induced astrocytes (hiAstros) were generated from commercially available human induced pluripotent stem cell (hiPSC) lines which are registered in the European Human Pluripotent Stem Cell Registry (hPSCreg®) (https://hpscreg.eu/):<br>ISFi001-A, also known as HMGU1 or #1 (the sex of donor - male) generated by the iPSC core unit at the Helmholtz Zentrum München, Germany<br>ISFi002-A, also known as HMGU12 (the sex of donor - male) generated by the iPSC core unit at the Helmholtz Zentrum München, Germany<br>UKERi82a-R1-002, also known as UKERi006-A or ERF31E2 (the sex of donor - female) generated by the Universitätsklinikum Erlangen (UKER), Germany
Authentication	The results of authentication are provided by supplier/provider (hPSCreg®, see https://hpscreg.eu/) and based on PCR, FACS and immunocytochemical detection of pluripotent factors OCT4, SOX2, NANOG and Lin28 as well as in vitro direct differentiation into progenitor progeny of the three germ layers ectoderm, mesoderm and endoderm. Thus no further authentication was performed by investigators.
Mycoplasma contamination	According to the information of the supplier/provider site (see https://hpscreg.eu/), three hiPSC cell lines used in this study tested negative for mycoplasma contamination. Thus no further mycoplasma contamination test was performed by investigators.
Commonly misidentified lines<br>(See ICLAC register)	No commonly misidentified cell lines were used this study.
